# Evidence that neural information flow is reversed between object perception and object reconstruction from memory

**DOI:** 10.1038/s41467-018-08080-2

**Published:** 2019-01-14

**Authors:** Juan Linde-Domingo, Matthias S. Treder, Casper Kerrén, Maria Wimber

**Affiliations:** 1 School of Psychology & Centre for Human Brain Health (CHBH), University of Birmingham, Birmingham, B15 2TT UK; 2 Cardiff University Brain Research Imaging Centre (CUBRIC), Cardiff University, Cardiff, CF24 4HQ UK

## Abstract

Remembering is a reconstructive process, yet little is known about how the reconstruction of a memory unfolds in time in the human brain. Here, we used reaction times and EEG time-series decoding to test the hypothesis that the information flow is reversed when an event is reconstructed from memory, compared to when the same event is initially being perceived. Across three experiments, we found highly consistent evidence supporting such a reversed stream. When seeing an object, low-level perceptual features were discriminated faster behaviourally, and could be decoded from brain activity earlier, than high-level conceptual features. This pattern reversed during associative memory recall, with reaction times and brain activity patterns now indicating that conceptual information was reconstructed more rapidly than perceptual details. Our findings support a neurobiologically plausible model of human memory, suggesting that memory retrieval is a hierarchical, multi-layered process that prioritises semantically meaningful information over perceptual details.

## Abstract (web summary)

Little is known about how the reconstruction of a memory unfolds in time in the human brain. Here, the authors provide evidence that the process of reconstructing the memory of an object involves a reversal of the information flow involved in the actual perception of that object.

## Introduction

When Rocky Balboa goes back to his old gym in the movie Rocky V, the boxing ring and the feeling of the dusted gloves in his hands trigger a flood of vivid images from the past. Like in many other movies featuring such mnemonic flashbacks, the main character seems capable of remembering what the room looked like years ago, who was there at the time, and even an emotional conversation with his old friend and coach Mickey. Perceptual details like colours, however, are initially missing in the scene, like in a faded photograph, and only gradually saturate over time. This common way of depicting memories in pop culture nicely illustrates that the memories we bring back to mind are not unitary constructs, and also not veridical copies of past events. Instead, it suggests that remembering is a reconstructive process that prioritises more meaningful components of an event over other, more shallow aspects^1,2^. We here report three experiments that shed light onto the temporal information flow during memory retrieval. Once a reminder has elicited a stored memory trace, are the different features of this memory reconstructed in a systematic, hierarchical way?

Surprisingly little is known about the time course of memory recall, considering our vast knowledge about the information processing hierarchy during visual perception. Visual object recognition is generally assumed to progress from low-level perceptual features, processed in early visual areas, to increasingly higher levels of integration and abstraction along the inferior temporal cortex^3–8^. What if a mental representation is re-created from memory, without much external stimulation? Retrieving a scene from Rocky V will elicit semantic knowledge about the film (e.g. the actor being Sylvester Stallone), but also mental images that can include fairly low-level details (e.g. whether the scene was in colour or in grey scale). How the brain manages to bring back each of these features when reconstructing an event from memory remains an open question. The present series of experiments tested our central working hypothesis that the stream of information processing is reversed during memory reconstruction compared with the perception of an external stimulus.

Over the last years, multivariate neuroimaging methods have made it possible to isolate brain activity patterns that carry information about externally presented stimuli, but also about internally generated mnemonic representations. Importantly, it has been shown that parts of the neural trace that an event produces during its initial encoding are reinstated during its later retrieval^9–14^. Most studies focused on the reactivation of abstract information, including a picture’s category^11,13,14^ or the task context in which it was encoded^10^. Evidence also exists for the reactivation of low-level perceptual details in early visual areas^15,16^. Moreover, a growing literature using electrophysiological methods is beginning to shed light onto the timing of such reinstatement, typically demonstrating neural reactivation within the first second after a reminder^12,17–19^, and sometimes very rapidly^16,20^. However, because all existing studies focused on a single feature of a memory representation (e.g., its semantic category), the fundamental question whether memory reconstruction follows a hierarchical information processing cascade, similar to perception, has not been investigated.

We hypothesise that such a processing hierarchy does exist, and that the information flow is reversed during memory retrieval compared with perception. That is, based on the widely accepted idea that memory reconstruction depends on back-projections from the hippocampus to neo-cortex^21,22^, we expect that those areas that are anatomically closer to the hippocampus (i.e., high-level conceptual processing areas along the inferior temporal cortex) are involved in the reactivation cascade faster than relatively remote areas (i.e., low-level perceptual processing areas). Therefore, we assume that once a reminder has initiated the reactivation of an associated event, higher-level abstract features will be reconstructed before lower-level perceptual features, producing an inverse temporal order of processing compared with perception.

**Fig. 1:**
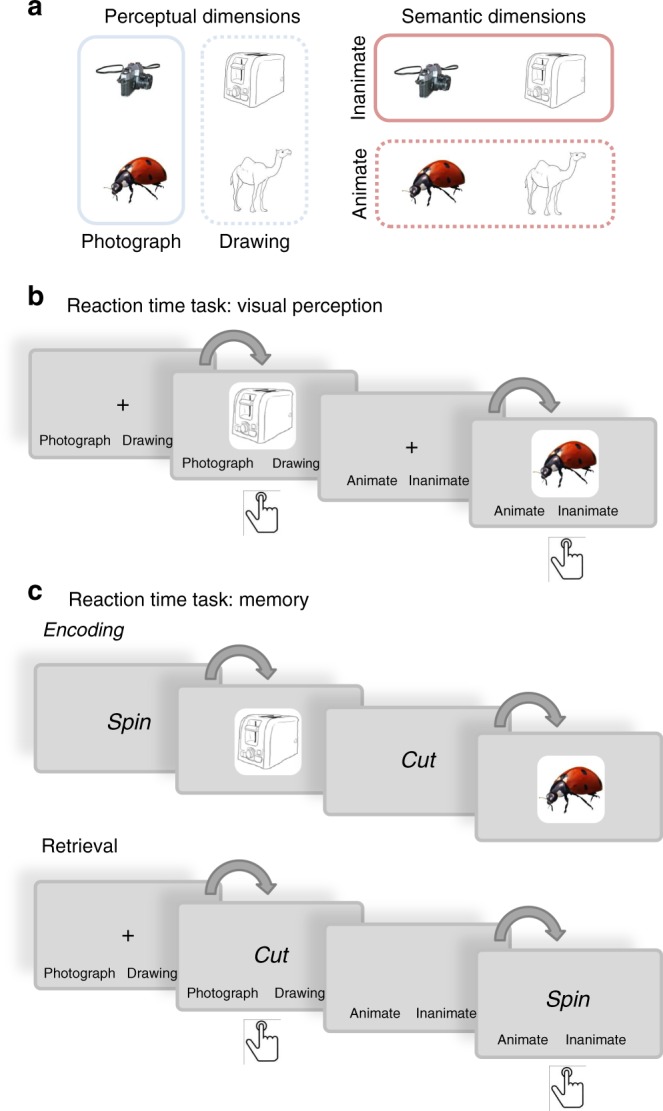
Stimuli and design of the behavioural experiments. **a** Illustration of the orthogonal design of the stimulus set. In all experiments, objects (a total of 128) varied along two dimensions: a perceptual dimension where objects could be presented as a photograph or as a line drawing; and a semantic dimension where objects could belong to the animate or inanimate category. **b** In the visual reaction time task, participants were prompted on each trial to categorise the upcoming object as fast as possible, either according to its perceptual category (photograph vs. line drawing) or its semantic category (animate vs. inanimate). **c** During the encoding phase of a memory reaction time task, participants were asked to create word-object associations (a total of eight per block). Reaction times were then measured during the retrieval phase, where subjects were presented with a reminder word, and asked to recall and categorise the associated object according to its perceptual (photograph vs. line drawing) or semantic (animate vs. inanimate) features. Button press symbols indicate at which moment in a trial RTs were collected

We tested this reverse reconstruction hypothesis in a series of two behavioural and one electroencephalography (EEG) experiment. All studies used a simple associative memory paradigm where participants learn arbitrary associations between word cues and everyday objects, and are later cued with the word to recall the object. In order to test for a processing hierarchy, it was important to independently manipulate the perceptual and conceptual contents of these objects. Therefore, objects varied along two orthogonal dimensions: one perceptual dimension, where the object was either presented as a photograph or a line drawing; and a semantic dimension where the object represents an animate or inanimate entity (Fig. 1a). The two behavioural experiments measure reaction times (RTs) while participants make perceptual or semantic category judgments for objects that are either visually presented on the screen, or reconstructed from memory. The EEG experiment uses a similar associative recall paradigm together with time-series decoding techniques^3,4,23^, allowing us to track at which exact moment in time perceptual and semantic components of the same object are reactivated, and to create a temporal map of semantic and perceptual features during perception and memory reconstruction. Our behavioural and electrophysiological findings consistently support the idea that memory reconstruction is not an all-or-none process, but rather progresses from higher-level semantic to lower-level perceptual features.

## Results

### Behavioural experiments

Our two behavioural experiments used RTs to test our central hypothesis that the information processing hierarchy reverses between the visual perception of an object and its reconstruction from memory. We assumed that the time required to answer a question about low-level perceptual features (photograph vs. drawing) compared to high-level semantic features (animate vs. inanimate) of an item reflects the speed at which these types of information become available in the brain. If so, reaction time patterns should reverse depending on whether the object is visually presented or reconstructed from memory: during perception, RTs should be faster for perceptual compared with semantic questions reflecting a forward processing hierarchy; during retrieval, RTs should be faster for semantic compared with perceptual questions if there is a reversal of that hierarchy.

Both experiments used a 2 × 2 mixed design (Fig. 1b, c), where all participants answered perceptual and semantic questions (factor question type, within-subjects) about the objects. Importantly, one group of participants was visually presented with the objects while answering these questions, whereas the other group recalled the objects from memory (factor task, between-subjects). The main difference between the two experiments was that in Experiment 1, both types of features were probed for each object; and in Experiment 2, objects were presented on background scenes (not of interest for the present purpose; see Methods section).

Overall accuracy in both experiments was near ceiling for the visual reaction time task (Experiment 1: *M* = 96.88%; SD = 2.40%; Experiment 2: *M* = 97.19%, SD = 2.99%), and high for the memory reaction time task (Experiment 1: 83.15%; SD = 0.92; Experiment 2: *M* = 66.23%, SD = 15.35%). Note that Experiment 2 was more difficult because participants had to memorise background scenes in addition to the objects’ semantic and perceptual features. In both experiments, only correct trials were used for all further RT analyses.

### RTs show the expected perception-to-memory reversal

To directly test for a reversal of the reaction time pattern between visual perception and memory reconstruction, we used generalised linear mixed-effect models (GLMM). GLMMs are ideal for modelling single trial (e.g. RT) data, without assumptions about the underlying distribution. They are able to capture variance explained by fixed and random variables, including the experimental manipulations of interest^24^. We used single trial RTs as target (dependent) variable. Fixed effects were the kind of task (visual vs. memory), question type (perceptual vs. semantic) and the interaction between task and question type. Participant IDs and slopes were included as random factors (including intercept).

Consistent with the reverse reconstruction hypothesis, we found that the interaction between task (visual vs. memory) and question type (perceptual vs. semantic) significantly predicted RTs in Experiment 1 (*F*_1,9020_ = 18.027, *P* < 0.001) and Experiment 2 (*F*_1,3280_ = 10.588, *P* = 0 .001). To test whether the interaction was produced by differences in the expected direction (perceptual < semantic during encoding, and semantic < perceptual during retrieval), planned comparisons were then performed for the visual and memory task independently, with question type as fixed effect. We found a significant effect of question type in the visual task (Experiment 1: *B* = −0.042, *t* = −3.973, *P* < 0.001; Experiment 2: *B* = −0.048, *t* = −2.457, *P* = 0.014), where the negative coefficient indicates that the model indeed predicted lower RTs for perceptual compared to semantic questions. A significant effect of question type was also found in the memory task, following the opposite pattern: positive coefficients now indicate significantly faster RTs during semantic than perceptual questions (Experiment 1: *B* = 0.156, *t* = 2.551, *P* = 0.011; Experiment 2: *B* = 0.165, *t* = 2.523, *P* = 0.012).

**Fig. 2:**
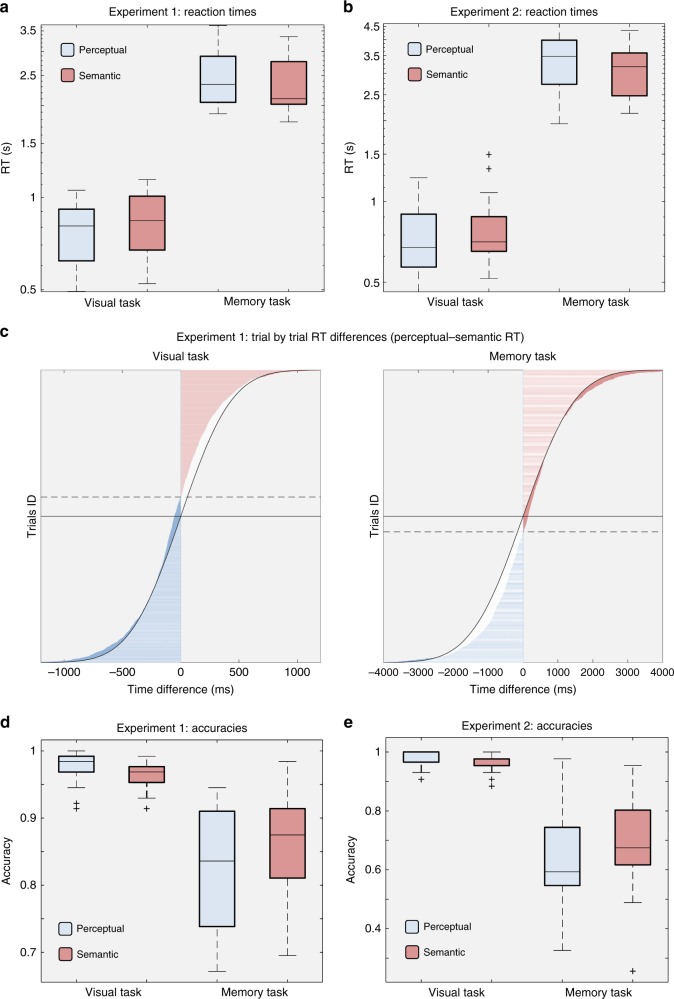
Behavioural RT and accuracy results. **a** Box plots representing reaction times in Experiments 1 and 2 (**b**) for perceptual (blue) and semantic (pink) questions when an object was physically presented on the screen (visual task, left) or cued by a reminder (memory task, right). We found that RTs were significantly predicted by an interaction between question type and kind of task (*P* < .001). For illustrative purposes the *Y*-axis in (**a**) and (**b**) is logarithmically scaled. **c** In Experiment 1, both types of questions were asked for each object representation. This allowed us to measure the difference in RTs between perceptual and semantic questions (*X*-axis) on a trial-by-trial level (*Y*-axis) during the visual task (left panel) and the memory task (right panel). Curved lines represent an expected normal distribution. The solid horizontal lines indicate the 50% point of the distribution (i.e., half of the trials), and dashed horizontal lines indicate the trial with a value closest to zero, where the perceptual–semantic difference is flipping from positive (pink) to negative (blue). If differences were normally distributed, the solid and dashed lines would be on top of each other. **d** Accuracy results in Experiment 1 for perceptual (blue) and semantic questions (pink) when the object was presented on the screen (visual task) or had to be recalled (memory task). Behavioural analyses showed that an interaction between type of task (i.e. visual or memory) and question type (i.e. perceptual or semantic) significantly predicted accuracy. **e** Box plots representing accuracy in Experiment 2 during the visual and memory task, where the significant interaction effect between type of task and question type was replicated. In all box plots, the line in the middle of each box represents the median, and the tops and bottoms of the boxes the 25th and 75th percentiles of the samples, respectively. Whiskers are drawn from the interquartile ranges to the furthest minimum (bottom) and maximum (top) values. Crosses represent outliers

For descriptive proposes, Fig. 2 also illustrates the distribution of participant-averaged RTs (Fig. 2a, b). During the visual task, participants on average were faster at answering perceptual (Experiment 1: *M* = 795 ms; SD = 235 ms; Experiment 2: *M* = 733 ms; SD = 211 ms) than semantic (Experiment 1: *M* = 842 ms, SD = 185 ms; Experiment 2: *M* = 797 ms, SD = 235) questions. When performing the same task on objects reconstructed from memory, they were now slower responding to perceptual (Experiment 1: *M* = 2502 ms; SD = 561; Experiment 2: *M* = 3348 ms, SD = 754) than semantic (Experiment 1: *M* = 2334 ms; SD = 534; Experiment 2: *M* = 3133 ms, SD = 660 ms) questions.

Reaction time analyses thus support our central hypothesis that the speed of information processing for different object features reverses between perception and memory, a pattern replicated between Experiments 1 and 2.

### Accuracies support a reversal between perception and memory

Next we investigated if a similar pattern was present in terms of accuracy (Fig. 2d, e). We used a GLMM with a logistic link function and a binary probability distribution for our target variable (accuracy, correct or incorrect on a given single trial). Fixed effects were the type of task (visual vs. memory), question type (perceptual vs. semantic), and the interaction between the two factors. Participant IDs and slopes were selected as random factors, including intercept.

In both experiments, the interaction between task (visual vs. memory) and question type (perceptual vs. semantic) significantly predicted participants’ accuracy (Experiment 1: *F*_1,11260_ = 12.215, *P* < 0.001; Experiment 2: *F*_1,4124_ = 8.383, *P* = 0.004). When running planned comparisons separately for the visual and the memory task in Experiment 1, results for the visual task revealed that question type significantly predicted accuracy (*F*_1,5886_ = 5.066, *P* = 0.024; *B* = −0.420, *t* = −2.251, *P* = 0.024), suggesting that accuracy for perceptual questions (*M* = 97.42%; SD = 2.68%) was higher compared to semantic questions (*M* = 96.33%; SD = 1.99%;). In the memory task, question type also predicted accuracy (*F*_1,5374_ = 5.374, *P* = 0.001; *B* = 0.251, *t* = 3.222, *P* = 0.001), with negative coefficients indicating that participants were more likely to give a correct answer in response to semantic (*M* = 85.83%; SD = 7.57%) than perceptual (*M* = 82.63%; SD = 8.79%) questions, in line with a reversed processing stream. Experiment 2 showed a similar trend in accuracy profiles. GLMM analyses for the visual task indicated that question type significantly predicted accuracy (*F*_1,2062_ = 4.371, *P* = 0.037; *B* = −0.585, *t* = −2.091, *P* = 0.037), with better performance for perceptual (*M* = 97.97%; SD = 2.77%) than semantic questions (*M* = 96.41%; SD = 3.07%). In contrast, for the memory task we found evidence for the prioritisation of higher-level information (semantic accuracy *M* = 69.57%; SD = 15.17%) over low-level details (perceptual accuracy *M* = 62.89%; SD = 15.09%). Here, question type again predicted accuracy in the expected direction (*F*_1,2062_ = 6.707, *P* = 0.010), with more accurate answers to semantic than perceptual questions (*B* = 0.319, *t* = 2.590, *P* = 0.010).

Altogether, the findings from our two behavioural experiments support our main hypothesis that during retrieval of a complex visual representation, the temporal order in which perceptual and semantic features are processed reverses compared with the initial perception. The results suggest that RTs can be used as a proxy to probe neural processing speed, as previously argued^25^. In the next sections, we report the findings from an EEG study that more directly taps into the neural processes that we believe are producing the behavioural pattern.

### EEG experiment

**Fig. 3:**
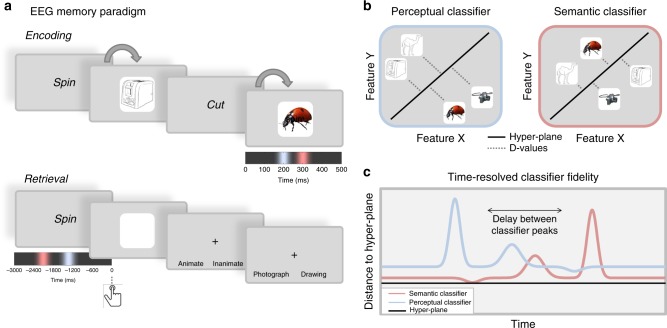
Design for EEG experiment and time-resolved multivariate decoding. In the EEG experiment participants were asked to create word-object associations (panel **a**), and to later reconstruct the object as vividly as possible when cued with the word, and to indicate with a button press when they had a vivid image back in mind. EEG was recorded during learning and recall, with the aim to perform time-series decoding analyses that can detect at which moment, within a single trial, a classifier is most likely to categorise perceptual and semantic features correctly. Coloured time lines under object and cue time windows represent our reversal hypothesis regarding the temporal order of maximum semantic (pink) and perceptual (blue) classification during the perception (encoding) and retrieval of an object. All EEG analyses were aligned to the object onset during encoding, and to the button press during retrieval. **b** Decoding analyses were performed independently per participant at each time point. For each given time point during a trial, two linear discriminant analysis (LDA)-based classifiers were trained on the EEG signal: one perceptual classifier discriminating photographs from line drawings, and one semantic classifier discriminating animate from inanimate objects. Classifiers were tested using a leave-one-out procedure, which allowed us to obtain a time series of confidence values (*d* values, reflecting the distance from the separation hyperplane) for each single trial. **c** Our main interest was to compare the time points of maximal fidelity of the perceptual (blue) and semantic classifiers (pink) on each trial, to test the hypothesis that the perceptual maximum (blue) precedes the semantic one (pink) during perception, and importantly that this order is reversed during memory recall

While the existing literature^25^ suggests that RTs tap into neural processing speed, we wanted to obtain a more direct signature of feature activation from brain activity. We therefore applied multivariate pattern analysis to EEG recordings, with the goal to pinpoint when in time, on an individual trial, the perceptual and semantic features of an object could be decoded from brain activity. We expected that perceptual information becomes available before semantic information when an object is visually presented on the screen, and expected the order of these peaks to reverse when the object is recalled from memory. The design closely followed the behavioural experiments, with the important difference that each participant now carried out a visual encoding phase that served to probe visual (forward) processing, and a subsequent recall phase used to probe mnemonic (backward) processing. The trial timing was optimised for obtaining a clean signal during object presentation and recall, rather than for RTs (Fig. 3). We therefore presented the perceptual and semantic questions only during the recall phase, and at the end of each trial, such that the questions would not bias processing towards perceptual or semantic features.

### Accuracy in the EEG study

In the retrieval phase of the EEG experiment, subjects were again cued with a word and asked to retrieve the associated object. They on average declared to retrieve the object on 93.60% of the trials (SD = 5.89%), with an average reaction time of 3046 ms (SD = 830 ms; minimum = 1369 ms; maximum = 5124 ms). We then asked two questions at the end of each trial, one perceptual and one semantic, which participants answered with an overall mean accuracy of 86.37% (SD = 6.6). Mirroring the behavioural experiments, average hit rates were 87.65% (SD = 6.57%) for semantic questions, and 85.08% (SD = 6.53%) for perceptual questions. A GLMM showed that the fixed factor question type predicted accuracy (*F*_1,5374_ = 7.706, *P* = 0.006), with perceptual questions showing a significantly lower hit rate than semantic questions (*B* = −0.225, *t* = −2.776, *P* = 0.006). Note that EEG participants were instructed to prioritise accuracy over speed, such that no meaningful RT measures could be obtained in this experiment.

### Evidence for a reversal in single-trial classifier fidelity

To determine the temporal trajectory of feature processing on a single trial level, we carried out a series of time-resolved decoding analyses. Linear discriminant analysis (LDA, see Methods section) was used to classify perceptual (photograph vs. drawing) and semantic (animate vs. inanimate) features of an object based on the EEG topography at a given time point, either during object presentation (encoding) or during object retrieval from memory (cued recall).

Our first aim was to confirm that there was a forward stream during encoding. Two separate classifiers were trained and tested to classify the perceptual (photograph vs. drawing) and the semantic category (animate vs. inanimate) of the to-be-encoded object, respectively, in each trial and time point per participant (see Fig. 3). Decoding was performed in separate time windows from 100 ms before stimulus to 500 ms post-stimulus. Our main interest was to determine the specific moment in each trial at which the perceptual and semantic classifiers showed the highest fidelity (Fig. 3b, c). For the encoding data, we thus identified the absolute *d* value peak per trial within 500 ms of stimulus onset. This approach allowed us to compare, within each trial, whether the classification peak for perceptual features occurred earlier than the peak for semantic features. Similarly, we used the cued recall time series to find the time points of maximum decodability of perceptual and semantic features during memory retrieval. Retrieval analyses are time-locked to the button press, i.e. the moment when participants declared that they retrieved the associated object from memory. The time window used in this analysis covered 3 s prior to participants’ responses, based on average RTs.

The first single-trial peak analysis was similar to the analysis conducted on RTs in the behavioural studies. A GLMM was used to test if the relative timing of *d* value peaks from the perceptual and semantic classifiers reverses between encoding and retrieval. The interaction between type of classifier and type of task significantly predicted the timing of *d* value peaks (*F*_1,5504_ = 8.632, *P* = 0.003). Planned comparisons between perceptual and semantic classifiers, run separately for encoding and retrieval, revealed that type of classifier did not significantly predict the timing of *d* value peaks during encoding (*F*_1,4326_ = 0.328, *P* = 0.567), but it did so during retrieval (*F*_1,1178_ = 3.879, *P* = 0.049). Beta coefficients showed that semantic peaks were predicted significantly earlier than perceptual peaks (*B* = 112.944, *t* = 1.969, *P* = 0.049), as expected if there is a reversed processing cascade.

**Fig. 4:**
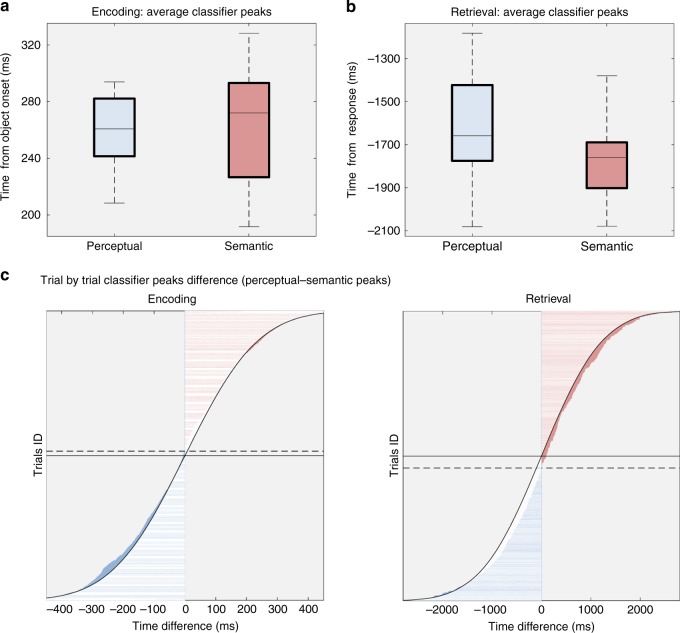
EEG multivariate analysis results. For illustrative purposes, box plots show group peak distributions of *d* values for perceptual and semantic categories during encoding (**a**; Perceptual peaks: *M* = 259 ms, SD = 24 ms; Semantic peaks: *M* = 267 ms, SD = 43 ms) and retrieval (**b**; Perceptual peaks: *M* = −1646 ms, SD = 247 ms; Semantic peaks: *M* = −1772 ms, SD = 177 ms) after averaging peaks within participants. All box plot elements represent the same metrics as in Fig. 2. **c** Measuring classifier fidelity in terms of *d* value peaks on a single-trial level allowed us to measure the pairwise time distance between perceptual and semantic peaks during encoding (left panel) and retrieval (right panel). *Y*-axis represents each individual trial, with trials accumulated across participants. The time distance between classifier peaks (time of perceptual peak minus time of semantic peak on a given trial) is represented on the *X*-axis. The curved line represents an expected normal distribution. The solid horizontal line indicates the 50% point (half of the trials), and the dashed horizontal line indicates the point where the temporal distance values change sign from perceptual < semantic (blue) to semantic < perceptual (pink)

We followed up this GLMM with a clustered Wilcoxon sign-rank test^26^ specifically analysing the relative order of semantic and perceptual peaks on each individual trial. At encoding (Fig. 4c), we found a significant difference (*T* = −9.764, *P* = 0.036) between the timing of perceptual and semantic peaks. Figure 4c shows that this difference was caused by a tendency of the single trial differences to be negative (leaning towards the blue side), suggesting that fidelity peaks for perceptual classification occurred earlier than those for semantic classification. This result validates our peak method, and confirms that low-level features are processed before high-level features during visual perception^3–6,8^. The results also suggest that an analysis that takes into account the paired difference between the classifier maxima from each single trial is more sensitive than a GLMM that uses the distributions of all single trials (not revealing a robust difference at encoding).

Importantly, following the same procedure, we next analysed the differences between the perceptual and semantic classifier peaks during memory reactivation, to test if the order reversed during retrieval compared with encoding. The single-trial approach ensured that the relative temporal order of perceptual and semantic peaks within a trial would be preserved even if the retrieval process was set off with varying delays across trials. A one-tailed clustered Wilcoxon signed rank test^26^, revealed a significant difference (*T* = 34.602, *P* < .001) when comparing perceptual with semantic *d* value peaks (leaning towards the red side in Fig. 4c). Critically, the one-tailed test in this case confirms our central hypothesis that during memory retrieval, semantic information can be classified in brain activity significantly earlier than perceptual information, suggesting that memory recall prioritises semantic over perceptual information.

### ERP results are consistent with a reversed processing

In a final step, we sought to corroborate our classifier-based findings by conventional event-related potential (ERP) analyses. If the differences picked up by the LDA classifier were produced by a signal that is relatively stable across trials and participants, these signal differences would also be visible in the average ERP time courses. A comparison of the ERP peaks during encoding and retrieval would then reveal the same perception-to-memory reversal as found in our multivariate analyses.

**Fig. 5:**
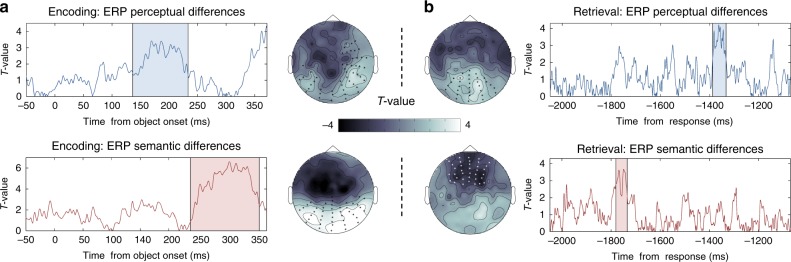
Univariate analysis results. **a** Left panels represent ERP group differences (*T* values) across time in those electrodes that formed a significant cluster during object presentation, locked to the onset of the stimulus. Top left panel shows the contrast of photographs vs. line drawings, and the bottom left panel differences between animate vs. inanimate objects. Scalp figures next to each contrast illustrate the maximum cluster’s topography, averaged across the significant time-window, with all significant electrodes in a cluster being marked with an asterisk. **b** Right panels show ERP group differences (*T* values) over time in those electrodes that are contained in the maximum significant clusters during memory retrieval, time locked to participants’ responses. The top right panel shows the perceptual contrast, and the bottom right panel the semantic contrast. Cluster topographies for each comparison are located next to each panel, and the temporal extent of significant clusters is shaded in colour

Firstly, a series of cluster-based permutation tests (see Methods) was performed during object presentation to test for ERP differences between perceptual and semantic categories. A perceptual contrast of the waveforms for photographs and line drawings revealed a significant positive cluster (*P*_corr_ = 0.008) between 136 ms and 232 ms after stimulus onset, with a maximum difference based on the sum of *T* values at 188 ms, and located over occipital and central electrodes (see Fig. 5a). Contrasting objects from the different semantic categories (animate and inanimate) revealed a later cluster over frontal and occipital electrodes (*P*_corr_ = 0.001) from 237 ms until 357 ms after stimulus presentation, with a maximum difference at 306 ms (see Fig. 5a). The peak semantic ERP difference for encoding thus occurred ~120 ms after the peak perceptual difference, consistent with the existing ERP literature^27^.

Similar contrasts between perceptual and semantic categories were then carried out during retrieval, again aligning trials to the button press. We found a significant perceptual cluster distinguishing the recall of photographs and line drawings over occipital electrodes (*P*_corr_ = 0.046) between 1390 and 1336 ms before participants’ responses, with a maximum difference at 1360 ms prior to response (see Fig. 5b). Comparing ERPs for the different semantic categories, we found a significant cluster distinguishing the recall of animate from inanimate objects over frontal electrodes (*P*_corr_ = 0.032) between 1781 and 1735 ms before object retrieval, with a maximum difference at −1770 ms (see Fig. 5b). Therefore, during memory retrieval, the peak semantic ERP difference occurred ~400 ms before the peak perceptual difference. Note that the timing of these effects is well aligned with the timing of the classifier results (see Fig. 4). Qualitatively, the ERP results thus mirror the results of our multivariate analyses, again supporting the reversal hypothesis.

An additional analysis was carried out to statistically test for an interaction on the ERP level between type of task (encoding vs. retrieval) and representational features (perceptual vs. semantic). In each participant, we identified the time point of the maximum ERP difference in each of our four comparisons of interest (i.e. photographs/drawings during encoding/retrieval; and animate/inanimate objects during encoding/retrieval). A 2 × 2 within-subjects ANOVA revealed a significant interaction between type of task and type of representational feature (*F*_1,42_ = 7.798, *P* = 0.011).

A final follow-up suggests that these ERP differences are not driven by a specific combination of perceptual and semantic features. For each of the clusters identified in the above ERP analysis, we ran a 2 × 2 within-subjects ANOVA, averaging the signal separately for the four types of sub-categories (animate-photographs, animate-line drawings, inanimate-photographs, inanimate-line drawings, see Supplementary Figure 1). We did not find a significant interaction between semantic and perceptual categories in any cluster during encoding (perceptual cluster: *F*_1,23_ = 1.106, *P* = 0.304; semantic cluster: *F*_1,23_ = 0.640, *P* = 0.432) or retrieval (perceptual cluster: *F*_1,20_ = 2.125, *P* = 0.160; semantic cluster: *F*_1,20_ = 0.403, *P* = 0.533), and thus no evidence indicating that our main ERP clusters were produced by a difference in one of the sub-categories that constitute the orthogonal dimension.

Altogether, the ERP results confirm that perceptual aspects are coded in brain activity earlier than semantic aspects during visual processing, but semantic differences dominate the EEG signal earlier than perceptual ones during retrieval.

## Discussion

How does the neural fingerprint of a memory unfold in time when triggered by a reminder? While it is widely accepted that visual object recognition starts with low-level perceptual followed by high-level abstract processing^3,4,6,8^, much less is known about the mnemonic feature processing cascade. Here we demonstrate that the reconstruction of a visual memory does depend on a hierarchical stream too, but this mnemonic stream follows the reverse order relative to visual processing. Across three experiments, we found highly converging evidence from RTs and accuracy (Experiments 1 and 2), multivariate classification analyses, and from univariate ERP analyses (Experiment 3), all indicating that conceptual information is prioritised during retrieval.

In the behavioural studies, participants were significantly faster at detecting low-level perceptual than abstract, conceptual differences during a visual classification task, while the object was presented on the screen. Critically, when probing the features of objects recalled from memory, the reverse effect was found: subjects required significantly less time to correctly retrieve semantic information about the object compared to perceptual details (see Fig. 2a, b). This reversal was corroborated by a significant interaction between the kind of feature (perceptual or semantic) and the kind of task (visual perception or memory recall task). Based on signal-detection models^28,29^, the RT findings suggest that during memory reconstruction, the decision threshold to identify abstract information of a mnemonic representation is reached before sufficient low-level information is available. The response latency pattern therefore supports our central hypothesis that the temporal order in which features come online is reversed when retrieving a stored representation of an object, relative to its perception. In addition to RTs, the same reversal pattern was present in accuracy profiles in both experiments (see Fig. 2d, e). These findings suggest a prioritisation of abstract semantic information over perceptual details of a mnemonic representation, consistent with hierarchical memory system models^30^.

The EEG results fully support the conclusions drawn from the behavioural studies. We used temporally resolved multivariate decoding analyses to observe when in time, during object perception and retrieval, the perceptual and semantic features of an object are maximally decodable from brain activity patterns. These analyses were carried out such that the relative temporal order of the perceptual and semantic classifier peaks could be directly compared in each single trial. When an object was visually presented during encoding, the maximum fidelity in classifying perceptual information (photograph vs. drawing) occurred ~100 ms earlier than the maximum for semantic information (animate vs. inanimate) (see Fig. 4a). This finding is consistent with a predominantly feed-forward processing as described previously^3–6,8^. Note that perceptual and semantic peaks during visual perception only differed statistically when comparing their relative timing on a single trial level, suggesting that such an analysis is more sensitive to detecting relatively small timing differences in noisy data. When we asked participants to reactivate an object’s representation from memory, semantic peaks were found ~300 ms earlier than perceptual peaks (see Fig. 4b). Like in the behavioural experiments, a consistent reversal between perception and memory was supported by a significant interaction between the type of feature that was probed (perceptual or semantic), and the type of task participants were engaged in (encoding or retrieval). Finally, we also found the same reversal pattern in the ERP peaks when comparing the maximum ERP difference between perceptual and semantic object classes. During object perception, the largest perceptual ERP cluster occurred ~100 ms before the semantic ERP cluster, whereas during retrieval the perceptual cluster followed the semantic one with a lag of about 400 ms (see Fig. 5). In summary, our results provide robust evidence for our main prediction that semantic features are prioritised over perceptual features during memory recall, in the opposite direction of the well-known forward stream of visual-perceptual processing. Follow-up studies will need to test whether this reversed stream is robust under different conditions, for example in tasks that explicitly vary the encoding demands to emphasise perceptual over semantic aspects of an event. If semantic information is always prioritised, this would suggest a hardwired characteristic of the output pathways from the hippocampus back to neocortex. Alternatively, and maybe more likely, the retrieved representation will to some degree also depend on what Marr^22^ called the “internal description” of a stimulus during encoding, including the rememberer’s goals and attentional state.

In our studies, the behavioural data were acquired separately from the EEG data, in a setting that was optimised for measuring RTs. Studies simultaneously measuring RTs and neural activity suggest that a meaningful relationship exists between EEG classifier fidelity values and human behaviour. In line with signal detection models^28,29^, it has been argued that the distance between two or more categories in a neural representational space serves as decision boundary that guides behavioural categorisation^25^. For example, Carlson et al. ^31^ used fMRI-based activation patterns in late visual brain regions in an object animacy task. They found that the faster the RT on a given trial, the further away in neural space the object was represented relative to the boundary between semantic categories. Similarly, an MEG study^25^ showed that the decision values during time points of maximum decodability, derived similar to our EEG decoding peaks, were strongly correlated with RTs for visual categorisation. Both studies thus suggest that during object vision, single-trial decoding measures reflect a distance between categories in neural space that translates into behaviour. Our findings indicate that this brain–behaviour relationship extends to mental object representations during memory reconstruction.

How does the reverse reconstruction hypothesis fit with existing knowledge about the neural pathways involved in memory reconstruction? It is generally accepted that during memory formation, information flows from domain-specific sensory modules via perirhinal and entorhinal cortices into the hippocampus. Recent evidence suggests that during visual processing, the coding of perceptual object information is preserved up to relatively late perirhinal processing stages^7^. The hippocampus is considered a domain-general structure^21,32,33^ whose major role is the associative binding of the various elements that constitute an episode^34–36^. The hippocampal code later allows a partial cue to trigger the reconstruction of these different elements from memory. This memory process likely depends on back-projections from the hippocampus to neocortical areas, causing the reactivation of memory patterns in (a subset of) the areas that were involved in perceiving the original event. Such reactivation has consistently been reported in higher-order sensory regions related to processing of complex stimulus and task information^10–12,14^, but also in relatively early sensory cortex^15,16^, suggesting that in principle, higher-level and lower-level information can be reconstructed from memory. Recent evidence, however, suggests that the structure of complex naturalistic events (movies) is transformed from perceptual to mnemonic codes during retrieval^9^. This finding is in line with the idea that remembering prioritises higher-order meaningful information over lower-level details.

While the reverse reconstruction hypothesis is neurobiologically plausible and has strong intuitive appeal, direct empirical evidence so far has been lacking. Indirect evidence comes from an fMRI study showing that within the medial temporal lobe, regions involved in visual object and scene processing are also activated when retrieving objects and scenes from memory, but with a delay relative to perception, consistent with a reversed information flow^37^. Intracranial EEG recordings have shown that connectivity between the entorhinal cortex and the hippocampus changes directionality between encoding and retrieval^38^, which could provide the functional basis for cortical reinstatement. Studies in rodents indicate that the hippocampus is in principle capable of replaying the neural code that represents a certain spatial memory in reverse order, in particular when the animal is awake and resting^39^. Finally, work using MEG-based decoding suggests that it is mainly the later visual processing stages that are reactivated during retrieval and mental imagery, consistent with a prioritisation of higher-level information^23,40^. Our proposal of a reverse processing hierarchy is thus plausible based on functional anatomy and the existing literature, even though it has never been explicitly tested so far.

We regard our reverse reconstruction hypothesis as complementary to existing models that address the nature and timing of different retrieval processes, including the influential dual process model (for a review see ref. ^41^). Dual process models focus on recognition rather than recall tasks, and on the cognitive processes and operations required to access a stored memory rather than the reactivated features of a memory. Successful recognition presumably can be based on a sense of familiarity, or on the recollection of contextual information from the initial encoding, an influential idea since the introspective analyses of William James^42^. While the original model does not explicitly address the time course of these processes, the EEG literature suggests that familiarity signals occur earlier (~300 ms) than recollection signals (starting from 500 to 600 ms)^43–46^. In contrast, all our experiments probed memory via cued recall, where successful recall strongly depends on the recollection of associative information. Our results suggest that within this recollection process, the semantic “gist” of a memory is accessed before perceptual details. Assuming that familiarity signals reflect a more gist-like and less detailed stage of the retrieval process than recollection signals (an assumption that some find controversial, see ref. ^47^), the hierarchical progression from an early global semantic signal to more fine-grained recollection might thus be a fundamental principle of retrieval that is shared between recall and recognition memory.

Interesting parallels also exist between our findings and visual learning phenomena like the Eureka effect^48^. The general idea that perception is shaped by stored representations has been proposed over a century ago by von Helmholtz^49^. A wealth of findings support the idea that previous exposures to a stimulus can exert a strong top-down influence on subsequent perception (for a review see ref. ^50^). Reminiscent of our present findings, Ahissar and Hochstein^51^ suggest that such visual learning is a top-down process that progresses from high-level to low-level visual areas. Specifically, they argue that improvements in visual discrimination (e.g. identifying a tilted line among distractors) are guided by high-level information (e.g. “the gist of the scene”) during earlier stages of learning, and increasingly by low-level information (e.g. line orientations or colours) at later stages. If abstract information is reactivated more easily during earlier stages of visual learning, it will influence performance more than detailed information. Even though speculative, the reverse reconstruction framework might thus have explanatory value for findings in related fields.

How our brain brings back to mind past events, and enriches our mental life with vivid images or sounds or scents beyond the current external stimulation, is still a fascinating and poorly understood phenomenon. Our results suggest that memories, once triggered by a reminder, unfold in a systematic and hierarchical way, and that the mnemonic processing hierarchy is reversed with respect to the major visual processing hierarchy. We hope that these findings can inspire more dynamic frameworks of memory retrieval that explicitly acknowledge the reconstructive nature of the process, rather than simply conceptualising memories as reactivated snapshots of past events. Such models will help us understand the heuristics and systematic biases that are inherent in our memories and memory-guided behaviours.

## Methods

### Participants

A total of 49 volunteers (39 female; mean age 20.02 ± 1.55 years) took part in behavioural Experiment 1. Twenty-six of them (19 female; mean age 20.62 ± 1.62 years) participated in the memory reaction time task. Five out of these 26 participants were not included in the final analysis due to poor memory performance (<66% general accuracy) compared with the rest of the group (*t*_24_ = 6.65, *P* *<* .01). Another group of 23 participants (20 female; mean age 19.35 ± 1.11 years) volunteered to participate in the visual reaction time task. In a second behavioural experiment (Experiment 2), 48 participants were recruited (42 female; mean age 19.25 ± .91 years). Twenty-four of them performed the memory reaction time task and another group of 24 took part in the visual reaction time task. For the electrophysiological experiment we recruited a total of 24 volunteers (20 female; mean age 21.91 ± 4.68 years). The first three subjects we recorded performed a slightly different task during retrieval blocks (i.e., they were not asked to mentally visualise the object for 3 s, and they had to answer only one of the perceptual and semantic questions per trial), and were therefore not included in any of the retrieval analyses. Since our paradigm was designed to test for a new effect, we did not have priors regarding the expected effect size. Behavioural piloting of the memory task showed a significant difference in RTs in a sample of *n* = 14. We therefore felt confident that the effect would replicate in our larger samples of *n* = 24 per group in each in the two behavioural experiments and the EEG experiment.

All participants reported being native or highly fluent English speakers, having normal (20/20) or corrected-to-normal vision, normal colour vision, and no history of neurological disorders. We received written informed consent from all participants before the beginning of the experiment. They were naïve as to the goals of the experiments, but were debriefed at the end. Participants were compensated for their time, receiving course credits or £6 per hour for participation in the behavioural task, or a total of £20 for participation in the electrophysiological experiment. The University of Birmingham’s Science, Technology, Engineering and Mathematics Ethical Review Committee approved all experiments.

### Stimuli

In total, 128 pictures of unique everyday objects and common animals were used in the main experiment, and a further 16 were used for practice purposes. Out of these, 96 were selected from the BOSS database^52^, and the remaining images were obtained from online royalty-free databases. All original images were pictures in colour on a white background. To produce two different semantic object categories, half of the objects were chosen to be animate while the other half was inanimate. Within the category of inanimate objects, we selected the same amount of electronic devices, clothes, fruits and vegetables (16 each). The animate category was composed of an equivalent number of mammals, birds, insects and marine animals (16 each). With the objective of creating two levels of perceptual manipulation, a freehand line drawing of each image was created using the free and open source GNU image manipulation software (www.gimp.org). Hence a total of 128 freehand drawings of the respective 128 pictures of everyday objects were created. Each drawing was composed of a white background and black lines to generate a schematic outline of each stimulus. For each subject, half of the objects were pseudo-randomly chosen to be presented as photographs, and half of them as drawings, with the restriction that the two perceptual categories were equally distributed across (i.e. orthogonal with respect to) the animate and inanimate object categories. All photographs and line drawings were presented at the centre of the screen with a rescaled size of 500 × 500 pixels. For the memory reaction time task and the EEG experiment, 128 action verbs were selected that served as associative cues. Experiment 2 also used colour background scenes of indoor and outdoor spaces (900 × 1600 pixels) that were obtained from online royalty-free databases, which are irrelevant for the present purpose.

### Procedure for Experiment 1—Visual reaction time task

Before the start of the experiment, participants were given oral instructions and completed a training block of four trials to become familiar with the task. The main perceptual task consisted of four blocks of 32 trials each (Fig. 1b). All trials started with a jittered fixation cross (500–1500 ms) that was followed by a question screen. On each trial, the question could either be a perceptual question asking the participant to decide as quickly as possible whether the upcoming object is shown as a colour photograph or as a line drawing; or a semantic question asking whether the upcoming object represents an animate or inanimate object. Two possible response options were displayed at the two opposite sides of the screen (right or left). The options for “animate” and “photograph” were always located on the right side to keep the response mapping easy. The question screen was displayed for 3 s, and an object was then added at the centre of the screen. In Experiment 2, this object was overlaid onto a background that filled large parts of the screen. Participants were asked to categorise the object in line with the question as fast as they could as soon as the object appeared on the screen, by pressing the left or right arrow on the keyboard. RTs were measured to test if participants were faster at making perceptual compared to semantic decisions.

All pictures were presented until the participant made a response but for a maximum of 10 s, after which the next trial started. Feedback about participants’ performance was presented at the end of each experimental block. There were 256 trials overall, with each object being presented twice across the experiment, once together with a perceptual and once with a semantic question. Repetitions of the same object were separated by a minimum distance of two intervening trials. In each block, we asked the semantic question first for half of the objects, and the perceptual question first for the other half.

The final reaction time analyses only included trials with correct responses, and excluded all trials with an RT that exceeded the average over subjects by ±2.5 standard deviations (SDs).

### Procedure for Experiment 1—Memory reaction time task

The memory version was kept very similar to the visual reaction time task, but we now measured RTs for objects that were reconstructed from memory rather than being presented on the screen, and we thus had to introduce a learning phase first. At the beginning of the session, all participants received instructions and performed two short practice blocks. Each of the overall 16 experimental blocks consisted of an associative learning phase (eight word–object associations) and a retrieval phase (16 trials, testing each object twice, once with a perceptual and once with a semantic question). The associative learning and the retrieval test were separated by a distractor task. During the learning phase (Fig. 1c), each trial started with a jittered fixation cross (between 500 and 1500 ms) that was followed by a unique action verb displayed on the screen (1500 ms). After presentation of another fixation cross (between 500 and 1500 ms), a picture of an object was presented on the centre of the screen for a minimum of 2 s and a maximum of 10 s. Participants were asked to come up with a vivid mental image that involved the object and the action verb presented in the current trial. They were instructed to press a key (up arrow on the keyboard) as soon as they had a clear association in mind; this button press initiated the onset of the next trial. Participants were made aware during the initial practice that they would later be asked about the object’s perceptual properties, as well as its meaning, and should thus pay attention to details including colour and shape. Within a participant, each semantic category and sub-category (electronic devices, clothes, fruits, vegetables, mammals, birds, insects, and marine animals) was presented equally often at each type of perceptual level (i.e. as a photograph or as a line drawing). The assignment of action verbs to objects for associative learning was random, and the occurrence of the semantic and perceptual object categories was equally distributed over the first and the second half of the experiment in order to avoid random sequences with overly strong clustering.

After each learning phase, participants performed a distractor task where they were asked to classify a random number (between 1 and 99) on the screen as odd or even. The task was self-paced and they were instructed to accomplish as many trials as they could in 45 s. At the end of the distractor task, they received feedback about their accuracy (i.e., how many trials they performed correctly in this block).

The retrieval phase (Fig. 1c) started following the distractor task. Each trial began with a jittered fixation cross (between 500 and 1500 ms), followed by a question screen asking either about the semantic (animate vs. inanimate) or perceptual (photograph vs. line drawing) features for the upcoming trial, just like in the visual perception version of the task. The question screen was displayed for 3 s by itself, and then one of the verbs presented in the directly preceding learning phase appeared above the two responses. We asked participants to bring back to mind the object that had been associated with this word and to answer the question as fast as possible by selecting the correct response alternative (left or right keyboard press). If they were unable to retrieve the object, participants were asked to press the down arrow. The next trial began as soon as an answer was selected. At the end of each retrieval block, a feedback screen showing the percentage of accurate responses was displayed.

Throughout the retrieval test, we probed memory for all word–object associations learned in the immediately preceding encoding phase in pseudorandom order. Each word–object association was tested twice, once together with a semantic and once with a perceptual question, with a minimum distance of two intervening trials. In addition, we controlled that the first question for half of the associations was semantic, and perceptual for the other half. Like in the visual RT task, the response options for “animate” and “photograph” responses were always located on the right side of the screen. In total, including instructions, a practice block and the 16 learning-distractor-retrieval blocks, the experiment took ~60 min.

For RT analyses we only used correct trials, and excluded all trials with an RT that exceeded the average over subjects by ±2.5 SDs.

### Procedure for Experiment 2—Visual reaction time task

Experiment 2 was very similar in design and procedures to Experiment 1, and we therefore only describe the differences between the two experiments in the following.

The second experiment started with a familiarisation phase where all objects were presented sequentially. In each trial of this phase, a jittered fixation cross (between 500 and 1500 ms) was followed by one screen that showed the photograph and line drawing version of one object simultaneously, next to each other. During the presentation of this screen (2.5 s) participants were asked to overtly name the object. After a jittered fixation cross (between 500 and 1500 ms), the name of the object was presented.

After this familiarisation phase, the experiment followed the same procedures as the visual reaction time task in Experiment 1 except for the following changes. Objects were overlaid onto a coloured background scene (1600 × 900 pixels). Also, each object (286 × 286 pixels) was probed only once, either together with a perceptual question, a semantic question (like above), or a contextual question asking whether the background scene was indoor or outdoor. For the current purpose we only describe the RTs to object-related questions in the Results section. Another minor difference to Experiment 1 was that in this version of the task, the question screen was displayed for 4 s, and the two options to answer during stimulus presentation were removed from the screen as soon as the reminder appeared.

### Procedure for Experiment 2—Memory reaction time task

The memory reaction time task in Experiment 2 also included, during the associative learning phase, a background scene (1600 × 900 pixels) that was shown on the screen behind each object (286 × 286 pixels), and participants were asked to remember the word–background–object combination. In this version of the task, each word–object association was tested only once, together with either a perceptual question about the object, a semantic question about the object, or a contextual question regarding the background scene (indoor or outdoor). Therefore, one-third of the objects were tested with a semantic question, one-third with a perceptual question, and one-third with a contextual question. Again, context was not further taken into account in the present analyses.

### Procedure for Experiment 3—EEG

Following the EEG set-up, instructions were given to participants and two blocks of practice were completed. The task procedure of the EEG experiment was similar to the memory task in Experiments 1 and 2 except for the retrieval phase (Fig. 3a). Each block started with a learning phase where participants created associations between overall eight action verbs and objects. After a 40 s distractor task, participants’ memory for these associations was tested in a cued recall test. In total, the experiment was composed of 16 blocks of eight associations each.

Each trial of the retrieval test started with a jittered fixation cross (500–1500 ms), followed by the presentation of one of the action verbs presented during the learning phase as a reminder. Participants were asked to visualise the object associated with this action verb as vividly and in as much detail as possible while the cue was on the screen. To capture the moment of retrieval, participants were asked to press the up-arrow key as soon as they had the object back in mind; or the down-arrow if they could not remember the object. This reminder was presented on the screen for a minimum of 2 s and until a response was made (maximum 7 s). Immediately afterwards, a blank square with the same size as the original image was displayed for 3 s. During this time, participants were asked to “mentally visualise the originally associated object on the blank square space”. After a short interval where only the fixation cross was present (500–1500 ms), a question screen was displayed for 10 s or until the participant's response, asking about perceptual (photograph vs. line drawing) or semantic (animate vs. inanimate) features of the retrieved representation, like in the behavioural tasks. However, in this case both types of questions were always asked on the same trial, and they were asked at the end of the trial rather than before the appearance of the reminder. The first question was semantic in half of the trials, and perceptual in the other half. Therefore, each retrieval phase consisted of eight trials where we tested all verb–object associations learned in the same block in random order.

### Data collection (behavioural and EEG)

Behavioural response recording and stimulus presentation were performed using Psychophysics Toolbox Version 3^53^ running under MATLAB 2014b (MathWorks). For response inputs we used a computer keyboard where directional arrows were selected as response buttons.

EEG data was acquired using a BioSemi Active-Two amplifier with 128 sintered Ag/AgCl active electrodes. Through a second computer the signal was recorded at a 1024 Hz sampling rate by means of the ActiView recording software (BioSemi, Amsterdam, the Netherlands). For all three experiments it was not possible for the experimenters to be blind to the conditions during data collection and analysis.

### GLMM analyses

Generalised linear mixed models (GLMMs) were used to test our alternative hypotheses for accuracy (all experiments), RTs (Experiments 1 and 2), and the relative timing of EEG classifier fidelity (*d* value) peaks (Experiment 3). We chose GLMMs instead of more commonly used GLM-based models (i.e., ANOVAs or *t*-tests) because they make fewer assumptions about the distribution of the data, are better suited to model RT-like data^24^ including our *d*-value peaks, and can accurately model proportional data that are bound between 0 and 1 (like memory accuracy). Our conditions of interest were modelled as fixed effects in the GLMM. Unless otherwise mentioned, these were the type of task (visual perception vs. memory retrieval) and the type of feature probed (perceptual vs. semantic). Our central reverse processing hypothesis was tested by an interaction contrast between the factors type of task and question type. Two further planned comparisons were then conducted to test if an interaction was driven by effects in the expected direction (e.g., RTs perceptual < semantic during visual perception, and semantic < perceptual during memory retrieval). For all analyses, participant ID (including intercept) was modelled as a random factor. Wherever possible, we also included slope as a random factor because GLMMs that do not take into account this factor tend to overestimate effects (that is, they are overly liberal^54^). In all cases, we used a compound symmetry structure based on theoretical assumptions and AIC and BIC values. We would like to emphasise that all of the effects reported as significant in the Results section remain significant (with a tendency for even stronger effects) when excluding the random factor slope, but we chose to report the results from the more conservative analysis.

Due to the data structure (specifically, the Hessian matrix not being positive definite), slope as a random effect could not be modelled in two of the analyses in Experiment 3: (i) when analysing the interaction between type of task and type of classifier as predictive factor for EEG classifier peaks; and (ii) when testing behavioural accuracy. In these two cases, the results are reported for GLMMs that do not include slope as a random factor. For the interaction analysis in (i), we also had to apply a linear transformation to the data, because the *d*-values during encoding and retrieval (which are compared directly in the interaction contrast) differed too much in scale. Data was thus *z*-scored to avoid errors calculating the Hessian matrix, and a constant value of 1000 ms was added to each value to avoid negative values in our target variable.

For all accuracy analyses we used a binomial distribution with a logistic link function. All models for analysing RTs and *d* value peaks used a gamma probability distribution and an identity link function. The choice of a gamma distribution was justified because in all cases it fit our single trial distributions better than alternative models, for example inverse Gaussian or normal distributions (evidence from AIC and BIC available on request).

### Clustered Wilcoxon signed rank test

To compare the pairwise differences between perceptual and semantic *d* value peaks in each encoding or retrieval trial (Experiment 3), and test whether the median of these differences deviates from zero in the expected direction (that is, perceptual < semantic during encoding, and semantic < perceptual during retrieval), we used a one-tailed Wilcoxon signed rank test that clustered the data per participant, using random permutations (2000 repetitions). This analysis was run using the R package “clusrank”^26^.

### EEG pre-processing

EEG data was pre-processed using the Fieldtrip toolbox (version from 3 August, 2017) for MATLAB^55^. Data recorded during the associative learning (encoding) phase was epoched into trials starting 500 ms before stimulus onset and lasting until 1500 ms after stimulus offset. The resulting signal was baseline corrected based on pre-stimulus signal (−500 ms to onset). Retrieval epochs contained segments from 4000 ms before until 500 ms post-response. Since the post-response signal during retrieval will likely still contain task-relevant (i.e., object specific) information, we baseline-corrected the signal based on the whole trial. Both datasets were filtered using a low-pass filter at 100 Hz and a high-pass filter at 0.1 Hz. To reduce line noise at 50 Hz we band-stop filtered the signal between 48 and 52 Hz. The signal was then visually inspected and all epochs that contained coarse artefacts were removed. As a result, a minimum of 92 and a maximum of 124 trials remained per participant for the encoding phase, and a range between 80 and 120 trials per subject remained for retrieval. Independent component analysis was then used to remove eye-blink and horizontal eye movement artefacts; this was followed by an interpolation of noisy channels. Finally, all data was referenced to a common-average-reference (CAR).

### Time-resolved multivariate decoding

First, to further increase the signal to noise ratio for multivariate decoding, we smoothed our pre-processed EEG time courses using a Gaussian kernel with a full-width at half-maximum of 24 ms. Time-resolved decoding via LDA using shrinkage regularisation^56^ was then carried out using custom-written code in MATLAB 2014b (MathWorks). Two independent classifiers were applied to each given time window and each trial (see Fig. 3b): one to classify the perceptual category (photograph or line drawing) and one to classify the semantic category (animate or inanimate). In both decoding analyses, we used undersampling after artefact rejection (i.e. for the category with more trials we randomly selected the same number of trials as available in the smallest category). The pre-processed raw amplitudes on the 128 EEG channels, at a given time point, were used as features for the classifier. LDA classification was performed separately for each participant and time point using a leave-one-out cross-validation approach. This procedure resulted in a decision value (*d* value) for each trial and time point, where the sign indicates in which category the observation had been classified (e.g., − for photographs and + for line drawings in the perceptual classifier), and the value of *d* indicates the distance to the hyper-plane that divided the two categories (with the hyper-plane being 0). This distance to the hyper-plane provided us with a single trial time-resolved value that indicates how confident the classifier was at assigning a given object to a given category. In order to use the resulting *d* values for further analysis, the sign of the *d* values in one category was inverted, resulting in *d* values that always reflected correct classification if they had a positive value, and increasingly confident classification with increasingly higher values.

Our main intention was to identify the specific moment within a given trial at which each of the two classifiers showed the highest fidelity, and to then compare the temporal order of the perceptual and semantic peaks. We thus found the maximum positive *d* value in each trial, separately for the semantic and perceptual classifiers. The time window used for *d* value peak selection covered 3 s prior to participants’ response and, based on behavioural RTs, only trials with an RT ≥ 3 s were included (rejecting a total of 1459 trials on a group level). For all further analyses we only used peaks with a value exceeding the 95th percentile of the classifier chance distribution (see section on bootstrapping below), such as to minimise the risk of including meaningless noise peaks. The resulting output from this approach allowed us to track and compare the temporal “emergence” of perceptual and semantic classification within each single-trial. When a peak for a given condition did not exceed the 95th percentile threshold, we did not include the trial in further analyses. For encoding trials, including all participants, we excluded 1.77 per cent of the trials based on this restriction. In the case of retrieval trials, all maximum peaks found exceeded the value of the threshold. In addition to this single-trial analysis, we also calculated the average *d* value peak latency for perceptual and semantic classification in each participant to compare the two average temporal distributions. Note, however, that many factors could obscure differences between semantic and perceptual peaks when using this average approach, including variance in processing speed across trials, e.g. for more or less difficult recalls. We therefore believe that the single trial values are more sensitive to differences in timing between the reactivated features. We used these single trial classifier peaks as dependent variables in a GLMM to test for an interaction between two fixed effects: the type of feature (perceptual vs. semantic) and the type of task (encoding vs. retrieval). Significant interaction results were followed up by planned comparisons to test for a significant effect of feature (perceptual vs. semantic) separately for encoding (expecting an earlier timing of perceptual than semantic peaks) and retrieval (expecting an earlier timing of semantic than perceptual peaks). Clustered Wilcoxon signed rank tests were then carried out to further corroborate the relative timing of the single-trial classifier peaks.

### Generating an empirical null distribution for the classifier

Previous work has shown that the true level of chance performance of a classifier can differ substantially from its theoretical chance level that is usually assumed to be 1/number of categories^57–59^. A known empirical null distribution of *d* values would allow us to determine a threshold for considering only those *d* value peaks as significant whose values are higher than the 95th percentile of this null distribution. We generated such an empirical null distribution of *d* values by repeating our classifier analysis with randomly shuffled labels a number of times, and combined this with a bootstrapping approach, as detailed in the following.

As a first step, we generated a set of *d* value outputs that were derived from carrying out the same decoding procedure as for the real data (including the leave-one-out cross-validation), but using category labels that were randomly shuffled at each repetition. This procedure was carried out independently per participant. On each repetition, before starting the time-resolved LDA, all trials were randomly divided into two categories with the constraint that each group contained a similar number of photographs and line drawings, and approximately the same amount of animate and inanimate objects (the difference in trial numbers was smaller than 8%). The output of one such repetition per participant was one *d* value per trial and time-point, just as in the real analysis. This procedure was conducted 150 times per participant for object perception (encoding) and retrieval, respectively, with a new random trial split and random label assignment on each repetition. For each participant we thus had a total of 151 classification outputs, one using the real labels, and 150 using the randomly shuffled labels.

Second, to estimate our classification chance distribution for the random-effects (i.e., trial-averaged) peak analyses, we used the 151 classification outputs from all participants in a bootstrapping procedure^60^. On each of the bootstrapped repetitions, we randomly selected one of the 151 classification outputs (150 from shuffled labels classifiers and one from a real labels classifier) per participant, and calculated the *d* value group average based on this random selection for each given time point. The real data was included to make our bootstrapping analyses more conservative, since under the null hypothesis, the real classifier output could have been obtained just by chance. This procedure was repeated with replacement 10,000 times. To generate different distributions for the perceptual and semantic classifiers, we ran this bootstrapping approach two times: once where the real labels output from each subject came from the semantic classifier, and once where the real *d* values came from the perceptual classifier.

### Univariate ERP analysis

A series of cluster-based permutation tests (Monte Carlo, 2000 repetitions, clusters with a minimum of two neighbouring channels within the FieldTrip software) was carried out in order to test for differences in ERPs between the two perceptual (photograph vs. line drawing) and the two semantic (animate vs. inanimate) categories, controlling for multiple comparisons across time and electrodes. First, we contrasted ERPs during object presentation in the encoding phase in the time interval from stimulus onset until 500 ms post-stimulus. We then carried out the same type of perceptual and semantic ERP contrasts during retrieval, in this case aligning all trials to the time of the button press. We used the full time window from 3000 ms before until 100 ms after the button press, but we further subdivided this time window into smaller epochs of 300 ms to run a series of *T* tests, again using cluster statistics to correct for multiple comparisons across time and electrodes. For all four contrasts, we reported the cluster with the lowest *P* value.

We were mainly interested in the temporal order of the ERP peaks that differentiated between perceptual and semantic classes during encoding and retrieval. The above procedure resulted in four statistically meaningful clusters across subjects: one each differentiating perceptual categories during encoding, semantic categories during encoding, perceptual categories during retrieval, and semantic categories during retrieval. To statistically test for an interaction in this timing of these clusters, we extracted the time point of the maximum ERP difference for each individual participant, restricted to the electrodes showing an overall cluster effect but over the entire time window for encoding and retrieval. These time points were entered into a 2 × 2 within-subjects ANOVA with the factors type of feature (perceptual or semantic), and type of task (encoding or retrieval), with the only planned comparison in this analysis being the interaction contrast.

### Code availability

The custom code used in this study is available in 10.17605/OSF.IO/327EK.

## Supplementary information

Supplementary Information

Peer Review File

## Data Availability

The data and that support the findings of this study are in [10.17605/OSF.IO/327EK].
