# Supplementary material for: Evidence that neural information flow is reversed between object perception and object reconstruction from memory
Source: Nat Commun. 2019 Jan 14;10:179. doi: 10.1038/s41467-018-08080-2 (PMC6331625; doi:10.1038/s41467-018-08080-2)
Supplement: Supplementary file 1 — Supplementary Information [file 41467_2018_8080_MOESM1_ESM.pdf]

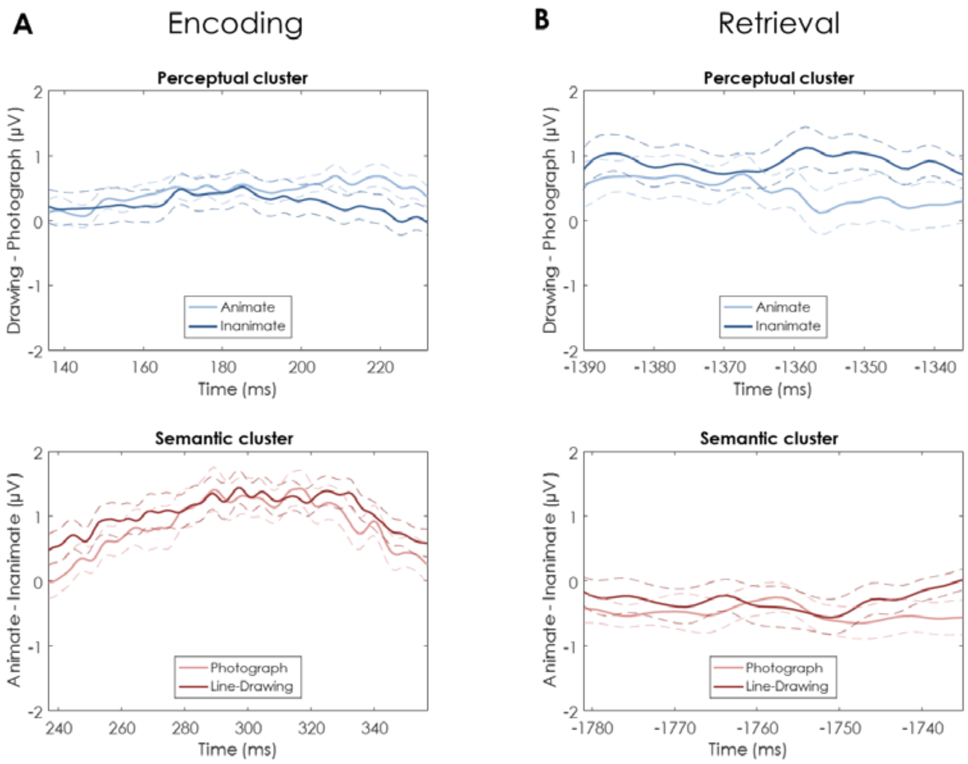

Supplementary Figure 1. Additional ERP results. Within the four significant ERP clusters reported in the main results (see Fig. 4), we did not find evidence suggesting any cluster was driven by a particular combination of perceptual and semantic features. (a) Upper panel: ERP differences between line drawings and photographs shown separately for animate (light blue) and in inanimate (dark blue) objects, based on same electrodes and time window contained in the main perceptual cluster found at encoding. Lower panel: ERP differences between animate and inanimate objects plotted separately for photographs (pink) and line drawing (dark red), based on the main semantic cluster found at encoding. (b) Upper panel: ERP differences between line drawings and photographs shown separately for animate (light blue) and in inanimate (dark blue) objects, based on same electrodes and time window contained in the main perceptual cluster found at retrieval. Lower panel: ERP differences between animate and inanimate objects plotted separately for photographs (pink) and line drawing (dark red), based on the main semantic cluster found at retrieval. In all four plots, dashed lines represented standard error of the mean. The results of a statistical comparison of the average T values are reported in the main results.
