# Supplementary material for: Evidence that neural information flow is reversed between object perception and object reconstruction from memory
Source: Nat Commun. 2019 Jan 14;10:179. doi: 10.1038/s41467-018-08080-2 (PMC6331625; doi:10.1038/s41467-018-08080-2)
Supplement: Supplementary file 2 — Peer Review File [file 41467_2018_8080_MOESM2_ESM.pdf]

Reviewers' comments:

Reviewer #1 (Remarks to the Author):

Review of Linde-Domingo et al., “Evidence for a reversal of the neural information flow between object perception and object reconstruction from memory”

During perception, information comes in through sense organs and flows up the cortical hierarchy, with the hippocampus sitting at the top of the hierarchy. It is widely assumed that episodic recollection runs in the reverse direction, such that retrieved information comes out of the hippocampus and flows down the cortical hierarchy. However, there is relatively little direct evidence in support of this view. In this paper, Linde-Domingo et al. test a concrete prediction arising from this view: namely, that during perception lower-level (perceptual) information will come online before higher-level (semantic) information, whereas during recall the opposite will be the case (semantic information will come online before perceptual information). This may seem like a fairly straightforward prediction, which makes it all the more important for it to be carefully tested, as the authors do here. Also, the study reinforces the foundational point that hippocampus is not primarily concerned with storing the “bitmap” of perceptual details; rather, it prioritizes the meaningful aspects of stimuli that are represented in brain areas that are closer (anatomically) to the hippocampus.

The authors muster convincing behavioral evidence (RTs and conditional accuracies) and EEG evidence (multivariate decoding and ERPs) in support of their hypothesis. My overall opinion of the paper is quite favorable -- while I have a few questions about the analysis methods, they should be easily addressable.

ANALYSIS QUESTIONS:

The authors mention the “important restriction” that they only used peaks with a value exceeding the 95th percentile of the classifier chance distribution. This brings up two issues: 1) What happens when the peak for a given condition (perceptual/semantic) for a given trial does not exceed this threshold? Is the trial thrown out? The authors should describe this more clearly and also state how many trials were thrown out. 2) Is this restriction necessary to get significant results? I would like to know what the results look like when the restriction is not included.

Why were the real data included in the null distribution (p. 23)?

COMMENTS ON THE DISCUSSION:

The paper (p. 15) says that the Chen et al. (2017, Nature Neuro) “Sherlock” paper shows that semantic structure of events is more consistently represented at recall (vs. perception) -- this is not totally accurate (e.g., Figure 4 from that paper shows that recall-recall classification is worse on average than movie-movie classification; there are some areas where recall-recall similarity is greater than movie-recall similarity -- that finding indicates a consistent “transformation” of codes from perception to memory, but it doesn’t show that semantic information is more reliably

represented during recall than during perception).

I don't see how this work is related to reverse replay of spatial sequences (as found, e.g., by Kurth-Nelson et al.). The paper (on p. 15) seems to be implying that backwards replay is an intrinsic feature of recall, but my understanding is that hippocampus can replay sequences both forwards and backwards (see, e.g., https://www.biorxiv.org/content/early/2018/01/03/225664).

On p. 16, the authors reference the finding that ERP correlates of familiarity come online before ERP correlates of recollection, and suggest that this fits with their results that semantic information comes online before perceptual information. The assumption that the FN400 signal is semantic is controversial -- I don't think that everyone would agree with it (see, e.g., Nyhus & Curran, 2009, Brain Research), so some hedging might be useful here.

Although this is by no means required, the authors could do a better job of making their code and data available online (e.g., by sharing code on github, or via new tools like codeocean.com that support running entire workflows in the cloud).

Signed,
Ken Norman (this narrative review constitutes my entire communication with the journal; as a matter of personal policy, I sign all of my reviews and I do not check any evaluative checkboxes on journal websites).

Reviewer #2 (Remarks to the Author):

Linde-Domingo et al present a set of interesting experiments investigating the order of processing and order of recall of perceptual and semantic information during simple object classification and recollection tasks. The central conceit is that the timecourse of information processing should essentially reverse during memory "reconstruction" when compared to "perception". I.e. perception should first reflect a forward cascade through low-level perceptual areas before contact is made with higher-level object representations, whereas "reconstruction" should show higher-level activation first, which then proceeds to low-level activation. This is investigated using a combination of behavioural measures and EEG. The approach is novel and the results have potential to be of substantial interest to the field. However, I have a number of misgivings about the solidity of the results that would need to be addressed for me to be convinced that the authors' conclusions are justified.

A consistent issue throughout the results is the lack of consideration of multiple comparisons. The authors report several significant interactions and follow these up with post-hoc t-tests. Correction for multiple comparisons is necessary, but is never mentioned. Such corrections may have substantial impact on the results. For example, pg 6, post-hoc tests on a significant interaction for 2 X 2 ANOVA are reported. There are 6 possible pairwise comparisons, of which only two are reported, and an alpha level of .05 is used. When performing post-hoc tests, you must consider all possible comparisons and correct the alpha level accordingly. The simplest such correction would be a

Bonferroni correction, which would give an alpha of ~.008. This would render the difference between perceptual and semantic questions in the memory task non-significant. Thus, for Experiment 1, the interpretation of the effect should be that there is a difference in RTs in the visual task but not in the memory task, not, as the authors report, that the effects *reverse*.

For the analysis of accuracies, it would seem to me that a generalized linear mixed model (GLMM) with a logistic link function would be a better approach here. Often using a standard ANOVA approach yields quite comparable results, but in this case you have some conditions near the boundary (90% +) and others in the middle (60-70%). The variance thus differs markedly (clearly seen in the SDs here), since with proportions the variance is dependent on the mean. This can sometimes lead to spurious interactions in ANOVAs (see Jaeger, 2008). Thus, here, where the interactions are absolutely critical to the main conclusions of the article, I think a GLMM is necessary.

Pg 6, lines 167-174. The authors perform Wilcoxon signed rank tests on the grounds that RTs are not necessarily normally distributed. Two things: 1) This seems to be due to a (fairly common) misconception. The tests themselves are being run on mean reaction times, not on the reaction times per se. Distributions of *mean* reaction times often *are* normally distributed, and it is these distributions on which the tests are run. The problem is really that the underlying RT distribution, which is what is being summarised by the mean, is usually skewed, and thus the mean is typically a poor summary of that distribution. It's better to consider using either median RTs or some transformation of the RTs (e.g. log or inverse transformations; see Ratcliff, 1993), which will have greater statistical power. 2) there's no need to do a Wilcoxon signed rank test here anyway unless the RTs are indeed not normally distributed, so report whether you checked if they are or not.

Pg 9. Lines 298-299. The results of a *one-tailed* Wilcoxon signed-rank test are what much of the paper hinges on. Using a one-tailed test does not seem terribly well justified. In principle, it should at least be possible for semantic effects to arise earlier than perceptual effects during encoding, even if this seems unlikely in practice. Use of a two-tailed statistics would make this comparison non-significant. However, a more important issue for me is that the authors run this test on all trials across all participants, as they believe this to be more sensitive (pg 22, lines 786-787). Any increase in sensitivity comes from ignoring non-independence in the data. Pairs of observations that come from one individual will tend to be correlated more than will pairs of observations across individuals. Each individual thus contributes a distinct cluster of observations that are not independent from each other. Ignoring this will typically underestimate the variance in the data and produce spuriously low p-values; given that the one-tailed p-value on pg 9 is only marginally significant in itself, this makes me cautious in accepting its validity. Indeed, the ANOVA based analysis, which does not suffer from this non-independence problem, does not find a significant effect at encoding. A better approach than either, might, again, be some kind of linear mixed effects analysis, which would account for the non-independence of observations, should have better statistical power than the ANOVA analysis, and could incorporate variability between objects into a single analysis.

Pgs 11-12. The mass univariate cluster-based permutation tests suffer from the absence of tests of interactions. The authors perform (twice, once for encoding, once for retrieval) two separate cluster based permutation tests, once contrasting the two perceptual categories, once contrasting the two

semantic categories. For encoding, they find a significant cluster between 136 ms and 232ms for the perceptual effect, and a significant cluster from 237ms to 357 ms for the semantic effect. On this basis they concluded that the semantic effect occurs later than the perceptual effect. However, that can't really be concluded from these tests alone. For that you need to test for an interaction. Suppose that the effect of perceptual category only holds for living objects. For living objects, drawings have a much higher amplitude than photographs. For non-living objects, there is no difference, and the amplitudes sit in-between the high effect of living drawings and low effect for living photographs. There would be a significant main effect of drawings versus photos, no significant effect of living versus non-living, and a significant interaction between the two. It would not make sense to interpret the main effect of perceptual category as if it were independent of semantic category. In other words, you need to demonstrate that there is no interaction, not simply that the effect is there in one condition and no the other. This is a variant on the issue described in "Erroneous analyses of interactions in neuroscience: a problem of significance"(Nieuwenhuis, Forstmann, & Wagenmakers, 2011) and by Gelman and Stern (2006).

Other points:

For the EEG classifiers: how many trials end up being entered into the analyses? It seems likely that there will be some trials with a significant peak for perceptual classification but no peak for semantic classification, and vice versa. How many trials were there with no significant peak for *either* classifier? Are the classifiers trained only on correct trials or on all trials?

Gelman, A., & Stern, H. (2006). The Difference Between "Significant" and "Not Significant" is not Itself Statistically Significant. The American Statistician, 60(4), 328–331. https://doi.org/10.1198/000313006X152649
Jaeger, T. F. (2008). Categorical data analysis: Away from ANOVAs (transformation or not) and towards logit mixed models. Journal of Memory and Language, 59(4), 434–446. https://doi.org/10.1016/j.jml.2007.11.007
Nieuwenhuis, S., Forstmann, B. U., & Wagenmakers, E.-J. (2011). Erroneous analyses of interactions in neuroscience: a problem of significance. Nature Neuroscience, 14(9), 1105–1107. https://doi.org/10.1038/nn.2886
Ratcliff, R. (1993). Methods for dealing with reaction time outliers. Psychological Bulletin, 114(3),

Reviewer #3 (Remarks to the Author):

NCOMMS-18-06568-T

Evidence for a reversal of the neural information flow between object perception and object reconstruction from memory

By Linde-Domingo et al.

This is a well-conceived study and well-written manuscript describing tests of the idea that the temporal order of perceptual and then semantic processing in visual perception are reversed during the retrieval from episodic memory of the same information. The authors present behavioral and neural (EEG) evidence in support of this hypothesis. They showed that RTs and accuracies patterns for perceptual vs. semantic information reversed from a visual task to a memory task. Also, neural evidence of 'peak responses' to these two types of information showed a similar result in that in the memory task the semantic information was decodable prior to the perceptual information (the revers of the visual task results). The authors do a nice job of motivating the study and situating it the perception/memory field more broadly. The use sophisticated but appropriate analyses for the EEG data, the results of which provide nice complements for the behavioral data.

I am largely convinced by these results and feel they offer a compelling empirical demonstration of an idea that has strong intuitive appeal. I've noted a few points that I would like to see addressed.

1) CONDITIONAL PROBABILITIES: In Fig 2c, it is not clear what these conditional probability scores of accuracy tell us. The raw accuracy scores for 'semantic' and 'perceptual' information already show greater accuracy for semantic vs perception. This might be driving the observed differences in conditional probabilities. This should be controlled, for example by accounting for the marginal probability of errors in each condition: e.g., P(sem/per)*P(per) and P(per/sem)*P(sem).

2) SINGLE TRIAL DIFFERENCES? The data in Fig 4 show distributions (and averages in c and d) of peak classifier scores for semantic and perceptual classifiers. These do not show WITHIN TRIAL differences between the peaks. The authors rightly note (stating line 783): "Note, however, that many factors could obscure differences between semantic and perceptual peaks when using this average approach, including variance in processing speed across trials, e.g. for more or less difficult recalls." and then "We therefore believe that the single trial values are more sensitive to differences in timing between the reactivated features." I agree with this conclusion, but I do not see this data in Fig 4 (unless I am misunderstanding what is being presented). This analysis needs to be done, or the description of the current analyses needs to be clarified so the reader understands this. For example, it may be more clear to calculate a time 'difference' between peak of S and P classifiers on EACH TRIAL and test that difference score for reliability across trials. Note: the authors claim on line 446 that Figs 4 c and d show the single trial data, but again, it is not clear this is the case.

3) RETRIEVAL PEAK IDENTIFICATION? The peaks for semantic and perceptual info in retrieval shown in Fig 5b appear quite different than the peaks in the encoding data (Fig 5a). There are obvious peaks in the encoding data, but the retrieval peaks are much noiser and variable in retrival. The peaks are much shorter and narrower. Is this really the best metric to evaluate the processing of semantic and perceptual information in these data? In the classifier data, the authors used '95th percentile of the chance distribution' to identify peaks in the data. There was no such control used to assess the univariate ERP peaks. This results in a quite unsatisfying and unconvincing analysis. For example, the perceptual peaks in the retrieval data (Fig 5b, top) show a number of early peaks that are wider than the identified 'peak' and nearly as tall, but appear much sooner, about -1800ms, the SAME time as the 'peak' of the semantic data. The operationalization of semantic and perception 'processing' from these data curves needs to be improved.

4) NULL DISTRIBUTION. What is the justification for only computing 50 shuffled-label classification scores for the null distribution? This is much lower than is typically used for generating null/chance distributions for classifier analysis.

MINOR

a) Rocky's old friend and coach was "Mickey" not "Michael".

b) line 204: "very late stage" is overkill. "after the retrieval of semantic information" or something like that would be more appropriate.

Response to the Reviewers
NCOMMS-18-06568-T

Linde-Domingo J, Kerrén C, Treder M, Wimber M

Reviewer #1 (Remarks to the Author):

During perception, information comes in through sense organs and flows up the cortical hierarchy, with the hippocampus sitting at the top of the hierarchy. It is widely assumed that episodic recollection runs in the reverse direction, such that retrieved information comes out of the hippocampus and flows down the cortical hierarchy. However, there is relatively little direct evidence in support of this view. In this paper, Linde-Domingo et al. test a concrete prediction arising from this view: namely, that during perception lower-level (perceptual) information will come online before higher-level (semantic) information, whereas during recall the opposite will be the case (semantic information will come online before perceptual information). This may seem like a fairly straightforward prediction, which makes it all the more important for it to be carefully tested, as the authors do here. Also, the study reinforces the foundational point that hippocampus is not primarily concerned with storing the "bitmap" of perceptual details; rather, it prioritizes the meaningful aspects of stimuli that are represented in brain areas that are closer (anatomically) to the hippocampus.

The authors muster convincing behavioral evidence (RTs and conditional accuracies) and EEG evidence (multivariate decoding and ERPs) in support of their hypothesis. My overall opinion of the paper is quite favorable -- while I have a few questions about the analysis methods, they should be easily addressable.

ANALYSIS QUESTIONS:

(1) The authors mention the "important restriction" that they only used peaks with a value exceeding the 95th percentile of the classifier chance distribution. This brings up two issues: 1) What happens when the peak for a given condition (perceptual/semantic) for a given trial does not exceed this threshold? Is the trial thrown out? The authors should describe this more clearly and also state how many trials were thrown out. 2) Is this restriction necessary to get significant results? I would like to know what the results look like when the restriction is not included.

Our use of the term "important" was clearly misleading, since the results remain the same irrespective of whether or not we use this more stringent threshold. In fact, the results from retrieval do not change at all because all the maximum peaks exceed the 95th percentile of the chance distribution. For encoding, the results slightly change to the better when not using this threshold: comparing the timing of all single trial perceptual and semantic peaks from encoding using a one-tailed Wilcoxon signed rank test, the effect we report in the manuscript when using the threshold (z = 1.87, P = 0.03) is still there, even more so, without the threshold (z = 2.06, P = 0.02). The same is true for the random effects analysis comparing the average peak latency without this restriction, where we report no significant difference in the manuscript (t_{20}= 0.67), and there is still no difference when not using the additional restriction (t_{20}= 0.71, P = .243). We would be happy to report the results without thresholding in the manuscript if the reviewer sees a merit, but otherwise feel it is more appropriate to keep with our a priori agreed procedure.

We have re-written the corresponding part of the methods section to better explain our procedure (see l. 815), and the number of excluded trials are reported in the results in l. 821.

(2) Why were the real data included in the null distribution (p. 23)?

We included the real data to make our bootstrapping analyses more conservative, the argument being that under the null hypothesis, the real classifier output could also have been obtained just by chance. Given that we now increased the number of shuffled-label classifiers from 50 to 150 (to address Reviewer #3's point #4), whether or not we include the real data does not change any of the results. We understand that this approach might be unusual, however, and would be happy to report the data without inclusion of the real classifiers if the reviewer finds this more appropriate. The rationale is now explained from l. 880 onwards of the manuscript.

COMMENTS ON THE DISCUSSION:

(3) The paper (p. 15) says that the Chen et al. (2017, Nature Neuro) "Sherlock" paper shows that semantic structure of events is more consistently represented at recall (vs. perception) -- this is not totally accurate (e.g., Figure 4 from that paper shows that recall-recall classification is worse on average than movie-movie classification; there are some areas where recall-recall similarity is greater than movie-recall similarity -- that finding indicates a consistent "transformation" of codes from perception to memory, but it doesn't show that semantic information is more reliably represented during recall than during perception).

We agree that this statement did not accurately reflect the Chen et al. paper, and have corrected the corresponding section of the discussion (l. 509).

(4) I don't see how this work is related to reverse replay of spatial sequences (as found, e.g., by Kurth-Nelson et al.). The paper (on p. 15) seems to be implying that backwards replay is an intrinsic feature of recall, but my understanding is that hippocampus can replay sequences both forwards and backwards (see, e.g., https://www.biorxiv.org/content/early/2018/01/03/225664).

We agree and have removed this particular reference from the discussion. We also qualify our main conclusion with respect to the rodent replay literature, emphasizing that reverse replay tends to occur relatively more frequently during "online" awake states, and it has been argued that these states might involve some degree of active retrieval in animals.

(5) On p. 16, the authors reference the finding that ERP correlates of familiarity come online before ERP correlates of recollection, and suggest that this fits with their results that semantic information comes online before perceptual information. The assumption that the FN400 signal is semantic is controversial -- I don't think that everyone would agree with it (see, e.g., Nyhus & Curran, 2009, Brain Research), so some hedging might be useful here.

We thank the reviewer for pointing out this controversy, which we now explicitly mention on p. 16, l. 546.

(6) Although this is by no means required, the authors could do a better job of making their code and data available online (e.g., by sharing code on github, or via new tools like codeocean.com that support running entire workflows in the cloud).

We fully agree and are currently in the process of preparing our code and data for online sharing together with the final manuscript.

Signed,
Ken Norman (this narrative review constitutes my entire communication with the journal; as a matter of personal policy, I sign all of my reviews and I do not check any evaluative checkboxes on journal websites).

Reviewer #2 (Remarks to the Author):

Linde-Domingo et al present a set of interesting experiments investigating the order of processing and order of recall of perceptual and semantic information during simple object classification and recollection tasks. The central conceit is that the timecourse of information processing should essentially reverse during memory "reconstruction" when compared to "perception". I.e. perception should first reflect a forward cascade through low-level perceptual areas before contact is made with higher-level object representations, whereas "reconstruction" should show higher-level activation first, which then proceeds to low-level activation. This is investigated using a combination of behavioural measures and EEG. The approach is novel and the results have potential to be of substantial interest to the field. However, I have a number of misgivings about the solidity of the results that would need to be addressed for me to be convinced that the authors' conclusions are justified.

(1) A consistent issue throughout the results is the lack of consideration of multiple comparisons. The authors report several significant interactions and follow these up with post-hoc t-tests. Correction for multiple comparisons is necessary, but is never mentioned. Such corrections may have substantial impact on the results. For example, pg 6, post-hoc tests on a significant interaction for 2 X 2 ANOVA are reported. There are 6 possible pairwise comparisons, of which only two are reported, and an alpha level of .05 is used. When performing post-hoc tests, you must consider all possible comparisons and correct the alpha level accordingly. The simplest such correction would be a Bonferroni correction, which would give an alpha of ~.008. This would render the difference between perceptual and semantic questions in the memory task non-significant. Thus, for Experiment 1, the interpretation of the effect should be that there is a difference in RTs in the visual task but not in the memory task, not, as the authors report, that the effects *reverse*.

We apologise for the misunderstanding, which we believe arose from our incorrect use of the term "post-hoc comparisons", where in reality we should have described these as "planned comparisons". Per design and hypotheses, apart from the critical interaction contrast, only 2 of all possible pair-wise comparisons are of interest in this study in order to demonstrate that the interaction is produced by effects in the expected direction: the difference between perceptual and semantic RTs at encoding (expected perceptual < semantic), and the difference between perceptual and semantic RTs at retrieval (expected semantic < perceptual). These are the relevant comparisons that were planned a priori, and are therefore reported in the manuscript.

Note that in the revised manuscript, we now follow the reviewer's suggestion to use GLMMs for all analyses involving single trials, and the 2x2 ANOVA results are no longer reported (see next points). It might be reassuring, however, to emphasize that all of the planned comparisons previously reported in our manuscript also survive an FDR correcting as implemented following https://ch.mathworks.com/matlabcentral/fileexchange/27418-fdr-bh.

(2) For the analysis of accuracies, it would seem to me that a generalized linear mixed model (GLMM) with a logistic link function would be a better approach here. Often using a standard ANOVA approach yields quite comparable results, but in this case you have some conditions near the boundary (90% +) and others in the middle (60-70%). The variance thus differs markedly (clearly seen in the SDs here), since with proportions the variance is dependent on the mean. This can sometimes lead to spurious interactions in ANOVAs (see Jaeger, 2008). Thus, here, where the interactions are absolutely critical to the main conclusions of the article, I think a GLMM is necessary.

We thank the reviewer for this excellent suggestion, and we agree that a GLMM is more appropriate for many of our analyses. Apart from correctly modelling the lower (i.e., 0) and upper (i.e., 1) boundaries of memory accuracy distributions, we also found that GLMM is more appropriate for modelling the single trial level data, and we therefore adopted this approach throughout the manuscript. The only exception is the ERP results, which are based on participant-averaged EEG signals and thus analysed using a traditional ANOVA. All new analyses fully confirm our initial hypotheses and can be found on p. 5, l. 139 (RT results in behavioural experiments), p. 6, l. 167 (accuracy results in behavioural experiments) and p. 10, l. 289 (classifier peak analysis).

(3) Pg 6, lines 167-174. The authors perform Wilcoxon signed rank tests on the grounds that RTs are not necessarily normally distributed. Two things: 1) This seems to be due to a (fairly common) misconception. The tests themselves are being run on mean reaction times, not on the reaction times per se. Distributions of *mean* reaction times often *are* normally distributed, and it is these distributions on which the tests are run. The problem is really that the underlying RT distribution, which is what is being summarised by the mean, is usually skewed, and thus the mean is typically a poor summary of that distribution. It's better to consider using either median RTs or some transformation of the RTs (e.g. log or inverse transformations; see Ratcliff, 1993), which will have greater statistical power. 2) there's no need to do a Wilcoxon signed rank test here anyway unless the RTs are indeed not normally distributed, so report whether you checked if they are or not.

We thank the reviewer for pointing out this misconception. We now analyse RT results using a GLMM, taking into account all single trials across the visual and the memory task. This analysis also tests for the critical interaction effect and appropriately models the between-subject variability; see our response to point #4. Importantly, GLMM are suited to model single trial data without assumptions about data distributions and data transformation is not needed (Lo & Andrews, 2015). Details about GLMM analyses are now reported in l. 764.

(4) Pg 9. Lines 298-299. The results of a *one-tailed* Wilcoxon signed-rank test are what much of the paper hinges on. Using a one-tailed test does not seem terribly well justified. In principle, it should at least be possible for semantic effects to arise earlier than perceptual effects during encoding, even if this seems unlikely in practice. Use of a two-tailed statistics would make this comparison non-significant. However, a more important issue for me is that the authors run this test on all trials across all participants, as they believe this to be more sensitive (pg 22, lines 786-787). Any increase in sensitivity comes from ignoring non-independence in the data. Pairs of observations that come from one individual will tend to be correlated more than will pairs of observations across individuals. Each individual thus contributes a distinct cluster of observations that are not independent from each other. Ignoring this will typically underestimate the variance in the data and produce spuriously low p-values; given that the one-tailed p-value on pg 9 is only marginally significant in itself, this makes me cautious in accepting its validity. Indeed, the ANOVA based analysis, which does not suffer from this non-independence problem, does not find a significant effect at encoding. A better approach than either, might, again, be some kind of linear mixed effects analysis, which would account for the non-independence of observations, should have better statistical power than the ANOVA analysis, and could incorporate variability between objects into a single analysis.

As indicated in our response to comment #3, we now use a GLMM also for the EEG classification data. The GLMM approach is explained on p. 22 l. 763 of the methods section, and the new results can be found from l. 289 onwards. The model, paralleling the one used for the revised RT analyses, takes into account all single trials, and includes fixed effects for the conditions of interest (type of classifier, type of task) and random effects (participant ID). The analysis shows that the classifier peaks are significantly predicted by an interaction between the type of classifier (perceptual vs

semantic) and the type of task (encoding vs retrieval), and this interaction has a p-value <.001, so is significant irrespective of whether we use one-tailed or two-tailed tests. As pointed out in our response to comment #3, we keep reporting the Wilcoxon tests in addition to the GLMM to specifically test for the pairwise difference in timing between perceptual and semantic peaks against an empirical null distribution. Note that this empirical distribution, obtained from shuffled-labels classifiers, also ignores the factor participant ID, and will produce the same spuriously low p-values as the real-label data. Since we had a clear directional hypothesis for these two planned comparisons (perceptual < semantic at encoding, and semantic < perceptual at retrieval), we believe it is justified to report one-tailed p-values here.

(5) Pgs 11-12. The mass univariate cluster-based permutation tests suffer from the absence of tests of interactions. The authors perform (twice, once for encoding, once for retrieval) two separate cluster based permutation tests, once contrasting the two perceptual categories, once contrasting the two semantic categories. For encoding, they find a significant cluster between 136 ms and 232ms for the perceptual effect, and a significant cluster from 237ms to 357 ms for the semantic effect. On this basis they concluded that the semantic effect occurs later than the perceptual effect. However, that can't really be concluded from these tests alone. For that you need to test for an interaction. Suppose that the effect of perceptual category only holds for living objects. For living objects, drawings have a much higher amplitude than photographs. For non-living objects, there is no difference, and the amplitudes sit in-between the high effect of living drawings and low effect for living photographs. There would be a significant main effect of drawings versus photos, no significant effect of living versus non-living, and a significant interaction between the two. It would not make sense to interpret the main effect of perceptual category as if it were independent of semantic category. In other words, you need to demonstrate that there is no interaction, not simply that the effect is there in one condition and no the other. This is a variant on the issue described in "Erroneous analyses of interactions in neuroscience: a problem of significance"(Nieuwenhuis, Forstmann, & Wagenmakers, 2011) and by Gelman and Stern (2006).

We include the requested analysis showing that there is no evidence for an interaction in the ERP clusters showing a main effect. The results are reported on p. 13 l. 385 of the main results, and illustrated in Supplementary Figure 1. For the two perceptual clusters (at encoding and at retrieval), we calculated the ERP difference between line drawings and photographs separately for animate and inanimate objects. Similarly, for semantic clusters, we calculate the animate vs inanimate ERP difference separately for photographs and line drawings. There was no evidence suggesting that the ERP differences in any of the four clusters (perceptual encoding, semantic encoding, perceptual retrieval, semantic retrieval) were driven by a particular combination of perceptual and semantic features.

Note that we also improved our statistical approach for the ERP analyses in general, in response to this comment as well as Reviewer #3's comment #3. Initially the ERP analyses were only meant to descriptively corroborate our classifier analysis, but we now support these findings with additional statistics. These new results testing for an interaction in ERP timing are reported on p. 12 L. 377.

Other points:

(6) For the EEG classifiers: how many trials end up being entered into the analyses? It seems likely that there will be some trials with a significant peak for perceptual classification but no peak for semantic classification, and vice versa. How many trials were there with no significant peak for *either* classifier? Are the classifiers trained only on correct trials or on all trials?

We thank the reviewer for pointing out this omission, also raised by Reviewer #1 (comment 1). The number of trials excluded due to the peak selection restriction, and a description of the results obtained without applying the threshold, can be found from l. 821 of the revised manuscript. For encoding, 1.77% of the trials were excluded based on this restriction. Specifically, these were 20 trials (across all participants) that had a perceptual but no semantic peak, and 19 trials that had a semantic but no perceptual peak. Including or excluding these trials does not change any of the results, and in fact most effects become slightly more pronounced without the threshold. In the case of retrieval, all maximum peaks exceeded the threshold so no trial had to be excluded.

Gelman, A., & Stern, H. (2006). The Difference Between "Significant" and "Not Significant" is not Itself Statistically Significant. The American Statistician, 60(4), 328–331. https://doi.org/10.1198/000313006X152649

Jaeger, T. F. (2008). Categorical data analysis: Away from ANOVAs (transformation or not) and towards logit mixed models. Journal of Memory and Language, 59(4), 434–446. https://doi.org/10.1016/j.jml.2007.11.007

Lo, S., & Andrews, S. (2015). To transform or not to transform: using generalized linear mixed models to analyse reaction time data. Frontiers in Psychology, 6(August), 1171. https://doi.org/10.1016/j.brainres.2009.05.091

Nieuwenhuis, S., Forstmann, B. U., & Wagenmakers, E.-J. (2011). Erroneous analyses of interactions in neuroscience: a problem of significance. Nature Neuroscience, 14(9), 1105–1107. https://doi.org/10.1038/nn.2886

Ratcliff, R. (1993). Methods for dealing with reaction time outliers. Psychological Bulletin, 114(3),

Reviewer #3 (Remarks to the Author):

This is a well-conceived study and well-written manuscript describing tests of the idea that the temporal order of perceptual and then semantic processing in visual perception are reversed during the retrieval from episodic memory of the same information. The authors present behavioral and neural (EEG) evidence in support of this hypothesis. They showed that RTs and accuracies patterns for perceptual vs. semantic information reversed from a visual task to a memory task. Also, neural evidence of 'peak responses' to these two types of information showed a similar result in that in the memory task the semantic information was decodable prior to the perceptual information (the revers of the visual task results). The authors do a nice job of motivating the study and situating it the perception/memory field more broadly. The use sophisticated but appropriate analyses for the EEG data, the results of which provide nice complements for the behavioral data.

I am largely convinced by these results and feel they offer a compelling empirical demonstration of an idea that has strong intuitive appeal. I've noted a few points that I would like to see addressed.

1) CONDITIONAL PROBABILITIES: In Fig 2c, it is not clear what these conditional probability scores of accuracy tell us. The raw accuracy scores for 'semantic' and 'perceptual' information already show greater accuracy for semantic vs perception. This might be driving the observed differences in conditional probabilities. This should be controlled, for example by accounting for the marginal probability of errors in each condition: e.g., P(sem/per)*P(per) and P(per/sem)*P(sem).

We thank the reviewer for this comment and, after running some simulations and additional analyses, we fully agree that the conditional probability results do not add any new information to the manuscript. As correctly noted by the reviewer, the differences in conditional probabilities are to a large degree driven by the significantly higher accuracies for semantic than perceptual questions. Unfortunately, the solution suggested by the reviewer does not solve this problem; in fact it nicely illustrates the point: P(sem/per) = P(sem∩per)/P(per), which multiplied with P(per) then results in P(sem∩per). Similarly, P(per/sem) = P(per∩sem)/P(sem), which when multiplied with P(sem) results in P(per∩sem). Therefore, both equations result in the intersection of answering perceptual and semantic questions correctly. We ran additional simulations, and tried different solutions, to make the conditional probabilities less dependent on baseline accuracies. None of these approaches resulted in any interesting effects, and for this reason we decided to exclude the conditional probability results from the revised manuscript.

2) SINGLE TRIAL DIFFERENCES? The data in Fig 4 show distributions (and averages in c and d) of peak classifier scores for semantic and perceptual classifiers. These do not show WITHIN TRIAL differences between the peaks. The authors rightly note (stating line 783): "Note, however, that many factors could obscure differences between semantic and perceptual peaks when using this average approach, including variance in processing speed across trials, e.g. for more or less difficult recalls." and then "We therefore believe that the single trial values are more sensitive to differences in timing between the reactivated features." I agree with this conclusion, but I do not see this data in Fig 4 (unless I am misunderstanding what is being presented). This analysis needs to be done, or the description of the current analyses needs to be clarified so the reader understands this. For example, it may be more clear to calculate a time 'difference' between peak of S and P classifiers on EACH TRIAL and test that difference score for reliability across trials. Note: the authors claim on line 446 that Figs 4 c and d show the single trial data, but again, it is not clear this is the case.

We thank the reviewer for pointing out this important gap in our figures and methods description. Following the reviewer's suggestion, we substituted Fig 4a and 4b with a new figure showing the trial by trial peak differences, rather than the overall distributions separately for perceptual and semantic

peaks. These pairwise differences in timing between perceptual and semantic peaks on each trial in fact constitute the input to our main analysis, where we test these differences against zero using a Wilcoxon signed rank test (see methods p. 24 l. 857, and results p. 10 l. 303). Note that in response to Reviewer #2's comments #3 and #4, we now also report the results of a GLMM that more accurately captures trial-specific and participant-specific variability in the timing of these peaks. A description of the GLMM approach can be found on p. 10 l. 289.

3) RETRIEVAL PEAK IDENTIFICATION? The peaks for semantic and perceptual info in retrieval shown in Fig 5b appear quite different than the peaks in the encoding data (Fig 5a). There are obvious peaks in the encoding data, but the retrieval peaks are much noisier and variable in retrieval. The peaks are much shorter and narrower. Is this really the best metric to evaluate the processing of semantic and perceptual information in these data? In the classifier data, the authors used '95th percentile of the chance distribution' to identify peaks in the data. There was no such control used to assess the univariate ERP peaks. This results in a quite unsatisfying and unconvincing analysis. For example, the perceptual peaks in the retrieval data (Fig 5b, top) show a number of early peaks that are wider than the identified 'peak' and nearly as tall, but appear much sooner, about -1800ms, the SAME time as the 'peak' of the semantic data. The operationalization of semantic and perception
'processing' from these data curves needs to be improved.

We fully agree that ERP results for retrieval data are noisier, as expected when assuming there is large variability in retrieval latency across participants and trials. For this reason, we believe that the best metric to test our specific hypothesis is the trial-by-trial, classifier-based analysis (see response to point #2). The ERP analysis in our previous manuscript was primarily meant to descriptively confirm the reversal hypothesis already shown in RTs and classifier peaks. The clusters shown in Fig. 5 are statistically meaningful in themselves, because they are obtained using FieldTrip's Monte-Carlo simulation to test for significant clusters. We do realise, however, that we never included statistics to test the temporal relationship between these clusters. We now report statistics to directly compare the timing of the different ERP peaks, testing for an interaction between type of task (encoding vs. retrieval) and representational features (perceptual vs. semantic), based on the timing of each single participant's average ERP peak. We found a significant interaction between type of task (encoding vs. retrieval) and type of feature comparison (i.e. perceptual vs. semantic) ($F_{1, 42}$ = 7.798, P = .011). These results are included in the revised manuscript from l. 377, and the approach is described in the methods section from l. 903.

4) NULL DISTRIBUTION. What is the justification for only computing 50 shuffled-label classification scores for the null distribution? This is much lower than is typically used for generating null/chance distributions for classifier analysis.

We thank the reviewer for prompting this important clarification. The 50 shuffled-label outputs do not in themselves constitute the null distribution, they only form the basis and first step in a bootstrapping procedure where a chance distribution is generated with 10,000 repetitions, randomly drawing one of the 50 shuffled-label outputs or the real classifier output per participant (as recommended by Stelzer, Chen, & Turner, 2013). This procedure is now more carefully explained from line 837. The main reason for us to compute only 50 shuffled outputs is that this procedure is computationally intense. In total, running the classifier using a leave-one-out approach, per participant and each second we trained a classier a total of ~102,400 times. Repeating this procedure using a random-label approach 50 times means running ~5,120,000 classifications, per second and participant.

However, in order to make sure that our chance distribution is indeed stable, we increased the number of shuffled-label outputs from 50 to 150 classifications per participant. This resulted in a new d-value threshold for selecting classifier peaks at encoding and retrieval. Using this new threshold led to the exclusion of one additional encoding trial (no changes at retrieval), and did not change any of the main results. All results are now updated using the new threshold based on 150 shuffled labels, and can be found from p. 9 l. 251.

MINOR

a) Rocky's old friend and coach was "Mickey" not "Michael".

He was of course called Mickey, now corrected (l. 26).

b) line 204: "very late stage" is overkill. "after the retrieval of semantic information" or something like that would be more appropriate.

We excluded the sentence in line with the reviewer's suggestion (from l. 174).

Reviewers' comments:

Reviewer #1 (Remarks to the Author):

The authors have fully addressed my concerns.
- Ken Norman

Reviewer #2 (Remarks to the Author):

In my previous review, I highlighted an issue with non-independence in the data, and using ANOVA with percentages, and suggested that the authors use GLMMs. I applaud the authors for taking this suggestion and going over and above it, employing GLMMs for RTs too. But unfortunately my concerns about the statistical support for their claims are not fully assuaged by the revision, because they still report and make claims based on the inappropriate Wilcoxon Signed-Rank test, which are not consistent with the more appropriate GLMM analysis. The GLMM analysis of the decoding data does not support the claim that decoding shows a difference in timing between perceptual and semantic information during encoding, and thus the title claim that there is a reversal of information flow between encoding and retrieval.
1) The results of the GLMM on the timing of peaks, which do appropriately respect clustering in the data, do not support the central claim that the timing of perceptual and semantic classifier peaks differs during encoding, although it does support the claim that these differ during retrieval. The results of the Wilcoxon signed-rank test, which is inappropriate for clustered data, do support that claim, but the test is not appropriate. This is not because of a “high number of degrees of freedom” (p24) – the Wilcoxon test does not involve degrees of freedom – but because data from within individuals is correlated. An alternative Wilcoxon signed-rank test that respects such clustering is available (for example, using the “clusrank” package for R), so there is no reason to use the inappropriate analysis.
2) The bootstrapping procedure for this analysis is also inappropriate for the same reason. You are still assuming that the clustering does not matter. Bootstrapping should attempt to mimic the data generating process – here you have trials which are clustered within individuals. So far as I can tell you permute within individuals only, but there are more appropriate ways to bootstrap with clustered data (e.g. http://biostat.mc.vanderbilt.edu/wiki/Main/HowToBootstrapCorrelat edData). A biased statistic still being significant when compared to a biased null distribution does not negate the fact they are both biased. The fact that a test that appropriately incorporates the clustered structure of the data does not find the same effect as an inappropriate test that does not suggests to me that the clustering is important and should not be ignored in this way.
3) It’s also still not clear to me why they’re using a Wilcoxon signed rank test in the first place, or why they’re deriving an empirical null distribution for it using bootstrapping; I can see why use bootstrapping for deciding which peaks are significant or not, but not for deciding whether the median of the distribution of those peaks differs from zero, which is what the WSR is doing.
4) Further to the bootstrapping, on pg 25 the authors talk of going a “random-effects” peak analysis on trial-averaged data etc, but it’s not clear to me what this relates to, as I can’t see such an analysis in the paper.
5) I still have to make a further request regarding the GLMMs. The authors use random-intercept

only models (i.e. they use participants as random-effects). Such models are prone to false-positives (Barr, Levy, Scheepers, & Tily, 2013), since they assume that effects are identical across participants and tend to underestimate standard errors. The authors should fit models with random slopes as well as random intercepts, in keeping with the suggestions of the Barr et al article. In my experience, fitting the maximal model (all possible random slopes) can be problematic with these kinds of data, so I just want to reassure the authors that I would be happy with a model that is as close to that as they can get.

Reviewer #3 (Remarks to the Author):

The authors were appropriately responsive to the reviews, and the manuscript has improved as a result.

Reviewer #1
We thank the reviewer for the positive evaluation and for the helpful input at earlier stages of the revision.

Reviewer #2

We thank the reviewer for these suggestions to further improve the manuscript. We followed the reviewer's advice in all respects, and we hope that the remaining statistical concerns are now adequately addressed.

1) The results of the GLMM on the timing of peaks, which do appropriately respect clustering in the data, do not support the central claim that the timing of perceptual and semantic classifier peaks differs during encoding, although it does support the claim that these differ during retrieval. The results of the Wilcoxon signed-rank test, which is inappropriate for clustered data, do support that claim, but the test is not appropriate. This is not because of a "high number of degrees of freedom" (p24) – the Wilcoxon test does not involve degrees of freedom – but because data from within individuals is correlated. An alternative Wilcoxon signed-rank test that respects such clustering is available (for example, using the "clusrank" package for R), so there is no reason to use the inappropriate analysis.

Following this suggestion, we implemented the clustered Wilcoxon test using the clusrank R package. The results fully support the conclusions previously drawn from the non-clustered test, with a significant difference between perceptual and semantic peaks at encoding (perceptual < semantic; T = -9.7642, P = .036) and at retrieval (semantic < perceptual; T = 34.602, P <.001). While the encoding effect is weaker than the retrieval effect, it is significant in the expected direction. Together with the RT and accuracy results from the two behavioural experiments and the ERP results, and based on the numerous existing studies using EEG or MEG during object processing, we are thus confident that a forward stream during encoding exists, and can be measured with a statistic that takes into account the single-trial differences between perceptual and semantic peaks. The new results can be found on p. 10 and 11, with the methods described on p. 23.

2) The bootstrapping procedure for this analysis is also inappropriate for the same reason. You are still assuming that the clustering does not matter. Bootstrapping should attempt to mimic the data generating process – here you have trials which are clustered within individuals. So far as I can tell you permute within individuals only, but there are more appropriate ways to bootstrap with clustered data (e.g. http://biostat.mc.vanderbilt.edu/wiki/Main/HowToBootstrapCorrelatedData). A biased statistic still being significant when compared to a biased null distribution does not negate the fact they are both biased. The fact that a test that appropriately incorporates the clustered structure of the data does not find the same effect as an inappropriate test that does not suggests to me that the clustering is important and should not be ignored in this way.

We understand the reviewer's concern regarding the bias in this statistic. While we believe that the bootstrapping procedure we originally used did in fact mimic the data generation

process, this analysis is no longer relevant given that we implemented the clustered Wilcoxon signed-rank test (see response to point 1 above) which does not need an additional baseline. The bootstrapping analysis and corresponding parts of the methods section have thus been removed.

3) It's also still not clear to me why they're using a Wilcoxon signed rank test in the first place, or why they're deriving an empirical null distribution for it using bootstrapping; I can see why use bootstrapping for deciding which peaks are significant or not, but not for deciding whether the median of the distribution of those peaks differs from zero, which is what the WSR is doing.

As mentioned in response to points 1) and 2), we now use the suggested clustered Wilcoxon test, and the bootstrapped baseline has been removed as a consequence.

To answer the question why we use the Wilcoxon in general: we believe it is important to include this data point, since it is the only statistical test in the manuscript that appropriately tests for paired differences between perceptual and semantic features on a single trial level (as shown in Figures 2 and 3). As argued in the manuscript (l. 322), a memory can be retrieved with a different offset on each trial, and such variations in retrieval latency will introduce noise across trials, but will not affect the ordering of semantic and perceptual features within a single trial. While the GLMM does take variance across trials into account, it does not pair perceptual and semantic RTs for each single trial, and does therefore not appropriately address our main hypothesis that this relative order within a trial remains the same. A Wilcoxon test is ideally suited to test exactly for such order effects, and we are thus keen on keeping these results in the manuscript.

4) Further to the bootstrapping, on pg 25 the authors talk of going a "random-effects" peak analysis on trial-averaged data etc, but it's not clear to me what this relates to, as I can't see such an analysis in the paper.

This description is no longer in the manuscript as a consequence of the changes in response to points 1-2.

5) I still have to make a further request regarding the GLMMs. The authors use random-intercept only models (i.e. they use participants as random-effects). Such models are prone to false-positives (Barr, Levy, Scheepers, & Tily, 2013), since they assume that effects are identical across participants and tend to underestimate standard errors. The authors should fit models with random slopes as well as random intercepts, in keeping with the suggestions of the Barr et al article. In my experience, fitting the maximal model (all possible random slopes) can be problematic with these kinds of data, so I just want to reassure the authors that I would be happy with a model that is as close to that as they can get.

We followed the reviewer's suggestion and fitted GLMMs that also include slope as a random effect. As anticipated, we ran into a number of difficulties using this approach. First, the models could not be estimated due to the complexity of the covariance structure. We

resolved this error by reducing the covariance to a simplified compound symmetry structure (following the guidance here http://www2.sas.com/proceedings/sugi30/198-30.pdf). The compound symmetry structure assumptions are plausible theoretically, and also supported empirically in our data (i.e., the best Arkaike and Bayesian Information Criteria were found for this structure). Using this compound symmetry covariance structure, the new model was able to estimate all effects (RTs and accuracy in Experiments 1 and 2, d-value peaks in Experiment 2) apart from the interaction contrast. All significant results previously reported in the manuscript remain significant. For 2 analyses (behavioural accuracy in Experiment 2, and the interaction for classifier peaks), slope could not be estimated within this new model because the Hessian matrix was not positive definite. For the interaction effect, we thus report the results for this effect based on a GLMM without slope as a random factor (but including participant ID and intercept as random factor). This is stated explicitly in the methods section (p.22). The old GLMM results are now replaced by these new results (across the whole results section, with an explanation in the methods p. 22). Note that the one effect that is not significant – as in the previously reported analyses - is the perceptual vs semantic difference during encoding when using GLMMs. As mentioned on p.10 of the results section, we believe that the single-trial (Wilcoxon) approach is more sensitive here because the peaks during encoding are very close together in time. To be transparent, the GLMM null result is now also explicitly discussed on p. 14 of the discussion. We believe, however, that combined with the existing literature on object processing using EEG or MEG, with our behavioural (RT and accuracy) findings, and with the ERP results, there is sufficient evidence for a forward stream during perception. The interaction and the reverse stream during retrieval are significant irrespective of the analysis approach across all experiments.

Overall, we hope that our new revisions sufficiently address the reviewer's concern, and we are grateful for the suggestions, which have helped to make the manuscript statistically more solid.

Reviewer #3
We thank the reviewer for the positive evaluation and for the helpful comments at earlier stages of the revision.

REVIEWERS' COMMENTS:

Reviewer #2 (Remarks to the Author):

The authors have responded as comprehensively and constructively as I've ever seen, really going the extra mile. And they'll be happy to hear I've nothing further to ask of them - I'm satisfied with what they've done.
